# Cultivation and characterization of *Candidatus* Nitrosocosmicus exaquare, an ammonia-oxidizing archaeon from a municipal wastewater treatment system

**DOI:** 10.1038/ismej.2016.192

**Published:** 2017-02-14

**Authors:** Laura A Sauder, Mads Albertsen, Katja Engel, Jasmin Schwarz, Per H Nielsen, Michael Wagner, Josh D Neufeld

**Affiliations:** 1 Department of Biology, University of Waterloo, Waterloo, Ontario, Canada; 2 Center for Microbial Communities, Department of Chemistry and Bioscience, Aalborg University, Aalborg, Denmark; 3 Department of Microbiology and Ecosystem Science, Division of Microbial Ecology, Research Network ‘Chemistry meets Microbiology’, University of Vienna, Vienna, Austria

## Abstract

*Thaumarchaeota* have been detected in several industrial and municipal wastewater treatment plants (WWTPs), despite the fact that ammonia-oxidizing archaea (AOA) are thought to be adapted to low ammonia environments. However, the activity, physiology and metabolism of WWTP-associated AOA remain poorly understood. We report the cultivation and complete genome sequence of *Candidatus* Nitrosocosmicus exaquare, a novel AOA representative from a municipal WWTP in Guelph, Ontario (Canada). In enrichment culture, *Ca.* N. exaquare oxidizes ammonia to nitrite stoichiometrically, is mesophilic, and tolerates at least 15 mm of ammonium chloride or sodium nitrite. Microautoradiography (MAR) for enrichment cultures demonstrates that *Ca*. N. exaquare assimilates bicarbonate in association with ammonia oxidation. However, despite using inorganic carbon, the ammonia-oxidizing activity of *Ca.* N. exaquare is greatly stimulated in enrichment culture by the addition of organic compounds, especially malate and succinate. *Ca.* N. exaquare cells are coccoid with a diameter of ~1–2 μm. Phylogenetically, *Ca.* N. exaquare belongs to the *Nitrososphaera* sister cluster within the Group I.1b *Thaumarchaeota*, a lineage which includes most other reported AOA sequences from municipal and industrial WWTPs. The 2.99 Mbp genome of *Ca.* N. exaquare encodes pathways for ammonia oxidation, bicarbonate fixation, and urea transport and breakdown. In addition, this genome encodes several key genes for dealing with oxidative stress, including peroxidase and catalase. Incubations of WWTP biofilm demonstrate partial inhibition of ammonia-oxidizing activity by 2-phenyl-4,4,5,5-tetramethylimidazoline-1-oxyl 3-oxide (PTIO), suggesting that *Ca.* N. exaquare-like AOA may contribute to nitrification *in situ*. However, CARD-FISH-MAR showed no incorporation of bicarbonate by detected *Thaumarchaeaota*, suggesting that detected AOA may incorporate non-bicarbonate carbon sources or rely on an alternative and yet unknown metabolism.

## Introduction

Nitrification is an important process for municipal and industrial wastewater treatment plants (WWTPs) because it prevents the negative impacts of releasing ammonia to receiving waters, including toxicity to fish, eutrophication and increased oxygen demand. Ammonia-oxidizing bacteria (AOB) were traditionally believed to mediate ammonia oxidation in soils, aquatic habitats and engineered environments. However, several studies have implicated recently discovered ammonia-oxidizing archaea (AOA) of the phylum *Thaumarchaeota* as the dominant ammonia oxidizers in many environments, including the open ocean (Wuchter *et al.*, 2006), soils (Leininger *et al.*, 2006; Stopnisek *et al.*, 2010; Yao *et al.*, 2011; Zhang *et al.*, 2012) and engineered environments such as aquaculture operations and aquarium biofilters (Sauder *et al.*, 2011; Brown *et al.*, 2013; Bagchi *et al.*, 2014). However, there is evidence that AOB are numerically dominant in some environments, and that AOB may mediate ammonia oxidation in several soils, despite a numerical dominance of AOA (Di *et al.*, 2009; Banning *et al.*, 2015; Sterngren *et al.*, 2015).

The role of AOA detected in WWTPs remains unclear. Compared with many natural environments, WWTPs contain relatively high levels of ammonia, which should favor AOB over AOA (Martens-Habbena *et al.*, 2009; Schleper, 2010). Indeed, many studies have reported a numerical dominance of AOB in municipal and industrial WWTPs (Wells *et al.*, 2009; Mussmann *et al.*, 2011; Gao *et al.*, 2013). Nevertheless, AOA have been detected in several WWTPs (Park *et al.*, 2006; Zhang *et al.*, 2009; Gao *et al.*, 2013, 2014), and in some cases outnumber AOB (Kayee *et al.*, 2011; Bai *et al.*, 2012). Although the abundance and diversity of AOA and AOB have been assessed in several WWTPs, no previous studies have analyzed the relative contributions of these groups to nitrification in any municipal WWTPs. Intact polar lipids originating from thaumarchaeol have been identified in WWTP biofilms (Sauder *et al.*, 2012), indicating that the detected *Thaumarchaeota* were viable in the system, though not necessarily oxidizing ammonia. Mussman *et al.* (2011) were unable to demonstrate bicarbonate assimilation by *Thaumarchaeota* in nitrifying sludge and called into question a strictly chemolithoautotrophic lifestyle of thaumarchaeotes in the examined industrial WWTP.

Although all cultured members of the *Thaumarchaeota* oxidize ammonia and fix inorganic carbon autotrophically, their metabolism in the environment may be more complex. AOA genomes encode transporters for a variety of organic compounds (for example, Hallam *et al.*, 2006; Walker *et al.*, 2010; Blainey *et al.*, 2011; Spang *et al.*, 2012) and archaea in the ocean incorporate amino acids (Ouverney and Fuhrman, 2000). In addition, supplementation of AOA cultures with pyruvate and alpha-ketoglutarate stimulates growth (Tourna *et al.*, 2011; Qin *et al.*, 2014), although this arises in several cultures from non-enzymatic detoxification of hydrogen peroxide (Kim *et al.*, 2016).

Although several Group I.1a *Thaumarchaeota* representatives have been reported in laboratory cultures (for example, Könneke *et al.*, 2005; Jung *et al.*, 2011; Mosier *et al.*, 2012; Lebedeva *et al.*, 2013; Li *et al.*, 2016), comparatively few Group I.1b AOA have been cultivated. Group I.1b representatives include *Nitrososphaera viennensis*, *Nitrososphaera evergladensis*, *Nitrosocosmicus franklandus* and *Nitrosocosmicus oleophilus*, which originate from soils or sediments (Tourna *et al.*, 2011; Zhalnina *et al.*, 2014; Lehtovirta-Morley *et al.*, 2016; Jung *et al.*, 2016), and *Nitrososphaera gargensis*, which originates from a hotspring (Hatzenpichler *et al.*, 2008; Palatinszky *et al.*, 2015). A phylogenetic analysis of archaeal *amoA* gene sequences demonstrated five major AOA lineages, represented by the genera *Nitrosopumilus*, *Nitrosocaldus*, *Nitrosotalea, Nitrososphaera*, and a fifth clade that forms a sister cluster to the *Nitrososphaera* (Pester *et al.*, 2012). This clade contains most *amoA* sequences obtained from WWTPs (for example, Mussmann *et al.*, 2011; Limpiyakorn *et al.*, 2011; Sauder *et al.*, 2012), manure composting facilities (Yamamoto *et al.*, 2010), landfill sites (Im *et al.*, 2011) and human skin (Probst *et al.*, 2013). Only one AOA enrichment culture exists from a wastewater treatment system, belonging to the Group I.1a *Thaumarchaeota* (Li *et al.*, 2016).

Here, we report the cultivation and characterization of a novel group I.1b *Thaumarchoaeta* representative, belonging to the *Nitrososphaera* sister cluster. This representative was enriched from biofilm of rotating biological contactors (RBCs) of a municipal WWTP in Guelph, Canada, where it was first discovered based on DNA and lipid signatures (Sauder *et al.*, 2012). We propose the name *Ca.* Nitrosocosmicus exaquare G61 for this representative.

## Materials and methods

### Sampling site

Biofilm for enrichment culture inoculation was collected from the Guelph WWTP in September 2012 from the 8th RBC (‘RBC 8’) of the Southeast (SE) treatment train. For a plant schematic and description of wastewater treatment processes, see Sauder *et al.* (2012). Biofilm for various experiments was collected at multiple time points; sampling details are summarized in Supplementary Table S1.

### Cultivation and experimental incubations

*Ca*. N. exaquare G61 was cultivated in a growth medium described previously (Krümmel and Harms, 1982; Hatzenpichler *et al.*, 2008), containing (per l): 0.05 g KH_2_PO_4_, 0.075 g KCl, 0.05 g MgSO_4_ × 7H_2_O, 0.58 g NaCl and 4 g CaCO_3_. After autoclaving, the medium was supplemented with filter-sterilized NH_4_Cl (0.5–1 mm), 1 ml selenite-tungstate solution and 1 ml trace element solution (Widdel and Bak, 1992; Könneke *et al.*, 2005). The resulting medium pH was ~8.5.

Culture incubations for generating a growth curve were performed in the described medium supplemented with 1 mm NH_4_Cl and 0.5 mm sodium pyruvate. At each sampling time, 2 ml culture was removed, pelleted for 10 min at 15 000 × *g*, and used for genomic DNA extractions. For temperature incubations, cultures were incubated with 0.5 mm NH_4_Cl at varying temperatures. For testing ammonia and nitrite tolerance, cultures were set-up with varying starting concentrations of NH_4_Cl and NaNO_2_, respectively. To test stimulation of *Ca.* N. exaquare by organic carbon, cells were subcultured (0.1% inoculum) and grown with or without organic carbon in the presence of 0.5 mm NH_4_Cl. Several organic carbon compounds were tested, including pyruvate, citrate, succinate, malate, acetate (final concentration 0.5 mm), glucose and taurine (0.25 mm), butyrate (0.1 mm), glycerol (0.0007%) and yeast extract (0.01%). All assays above were conducted in triplicate with a 1% inoculum, in the dark and without shaking.

### Inhibitor assays on WWTP biofilm

Biofilm and wastewater samples were obtained from the Guelph WWTP in April and December 2015 and were stored on ice until returned to the laboratory (~1 h). Incubations were performed in 125 ml glass serum bottles using 20 ml volumes. Incubations consisted of 2% (w/v) biofilm suspensions in 0.22-μm filtered RBC influent amended with 1 mm ammonium chloride. Flasks were supplemented with inhibitors as appropriate, including 6 μm acetylene (aqueous), 10 μm allylthiourea (ATU), 8 μm octyne (aqueous) and both 200 and 400 μm PTIO. Incubations were performed in triplicate, in the dark, without shaking.

### Water chemistry measurements

Ammonia and nitrite concentrations were measured colourimetrically according to previously published protocols using Nessler’s reagent (Meseguer-Lloret *et al.*, 2002) and Griess reagent (Miranda *et al.*, 2001), respectively. Absorbance values were measured at 550 nm (nitrite) and 450 nm (ammonia) using a Filtermax F5 Multi-Mode Microplate Reader (Molecular Devices, Sunnyvale, CA, USA). All technical measurements were performed in duplicate. Substrate concentrations were determined by comparison to standards using SoftMax Pro 6.4 (Molecular Devices). Note that for reported ammonia concentrations, values refer to total ammonia (NH_4_^+^+NH_3_), unless otherwise indicated.

### DNA extractions and quantitative PCR

Genomic DNA extractions were performed using the PowerSoil DNA Isolation Kit (for biofilm) or the Ultraclean Microbial DNA Isolation Kit (for laboratory cultures), according to the manufacturer’s instructions (MO BIO, Carlsbad, CA, USA). Beadbeating was performed using a FastPrep-24 (MP Biomedicals, Santa Ana, CA, USA) at 5.5 m s^−1^ for 45 s. Quantification of thaumarchaeotal and bacterial 16S rRNA genes was performed using primers 771F and 957R (Ochsenreiter *et al.*, 2003) and 341F and 518R (Muyzer *et al.*, 1993), respectively. AOB *amoA* genes were quantified using primers amoA1F and amoA2R (Rotthauwe *et al.*, 1997). All qPCR amplifications used SYBR Green Supermix (Bio-Rad, Hercules, CA, USA) and were performed using technical duplicates on a CFX96 system (Bio-Rad). All efficiencies were >80% and *R*^2^ values were >0.99. For growth curves, *Ca*. N. exaquare cell numbers were estimated based on measured 16S rRNA gene copies (assuming one chromosome per cell), divided in half to account for two 16S rRNA gene copies per genome. Generation time was estimated from the slope of the natural log-transformed thaumarchaeotal cell numbers during exponential growth.

### Phylogenetic analyses

*Ca.* N. exaquare *amoA* gene sequences were compared with cultivated AOA representatives and environmental sequences obtained from GenBank. Global alignment of sequences was performed using MUSCLE (Edgar, 2004). Evolutionary histories were inferred using the maximum likelihood method based on the general time reversible model of sequence evolution. A Gamma distribution was used to model evolutionary rate differences among sites. Bootstrap testing was conducted with 500 replicates. All alignments and phylogenetic analyses were conducted in MEGA6 (Tamura *et al.*, 2013).

### Microscopy

For scanning electron microscopy (SEM), cells were applied to a silicon wafer attached to an aluminum stub with conductive carbon tape, then imaged (unfixed and unstained) using a LEO 1550 field-emission scanning electron microscope (Zeiss, Oberkochen, DE, USA) with an InLens SE detector, an EHT of 7 kV and a working distance of 10.3 mm.

For catalyzed reporter deposition-fluorescence *in situ* microscopy (CARD-FISH), samples were fixed and processed as described previously (Ishii *et al.*, 2004; Mussmann *et al.*, 2011), with modifications. Briefly, cells were permeabilized with proteinase K (15 μg ml^−^^1^) for 10 min at room temperature, followed by proteinase K inactivation by 0.01 m HCl for 20 min. To inactivate endogenous peroxidases, slides were treated with 0.3% H_2_O_2_ in 100% methanol for 30 min (enrichment cultures) or overnight (biofilm). Probes Arch915 (Stahl and Amann, 1991) or Thaum726 (Beam, 2015) were used for detecting thaumarchaeotes, with formamide concentrations of 10% and 25%, respectively. Thaum726 was used with competitor probes thaum726_compA and thaum726_compB (Supplementary Table S2). For AOB and *Nitrospira*, previously published probe mixes were used (Supplementary Table S2). DAIME image analysis software (Daims *et al.*, 2006) was used for quantitative FISH (AOB and nitrite oxidizing bacteria (NOB)) and quantitative CARD-FISH (AOA) analyses.

### MAR-FISH

Incubations of *Ca*. N. exaquare were prepared in 5 ml volumes in 50 ml tissue culture flasks. Cells were incubated with 0.5 mm NH_4_Cl in HEPES-buffered freshwater medium (FWM; Tourna *et al.*, 2011), with calcium carbonate excluded to minimize unlabeled inorganic carbon availability. Each flask was amended with 10 μCi ^14^C-bicarbonate (Hanke Laboratory Products, Vienna, Austria). Flasks were incubated at 28 °C for 24 h in the dark, without shaking. For biofilm incubations, biofilm from SE RBC 1 and 8 was collected and chilled until experimental set-up. Biofilm suspensions were diluted 1:5 in RBC influent, and pre-incubated for 3 h with 0.05 mm NH_4_Cl at ambient temperature. Biofilm suspensions were then aliquoted into 5 ml volumes amended with 0.5 mm NH_4_Cl and 10 μCi ^14^C-bicarbonate (in duplicate). Flasks were incubated at ambient temperature in the dark, without shaking, and biomass was removed and fixed after 6 and 20 h of incubation. Biomass was fixed with 4% paraformaldehyde and CARD-FISH was performed as described above; MAR was performed as described previously (Lee *et al.*, 1999; Mussmann *et al.*, 2011). Enrichment culture samples were exposed for 12 days, and biofilm culture samples were exposed for 9, 12 and 15 days. Samples were imaged on a Leica TCS SP8 confocal laser scanning microscope (CLSM). When necessary, MAR signals were recorded with a color-CCD camera (DFC 450, Leica Microsystem, Wetzlar, Germany), attached to the CLSM, to differentiate between black silver grains and brown biofilm material.

### Sequencing, genome assembly and genome annotation

Genomic DNA for sequencing was extracted from *Ca.* N. exaquare using the PowerSoil DNA Isolation Kit (MO BIO Laboratories). Enrichment cultures containing either no organic carbon or supplemented with 0.5 mm taurine were extracted separately to generate metagenomes suitable for differential abundance binning.

Genomic DNA was prepared for sequencing using the TruSeq PCR-free kit (Illumina, San Diego, CA, USA) using alternative nebulizer fragmentation, gel-free size selection and a 550 bp target insert size. A mate-pair library was prepared with the Nextera Mate Pair Sample Preparation Kit (Illumina) and sequenced (2 × 301 bases) using MiSeq Reagent Kit v3 (Illumina). Paired-end FASTQ reads were imported to CLC Genomics Workbench version 7.0 (CLC Bio, Qiagen, Hilden, Germany) and trimmed using a minimum Phred score of 20 and length of 50 bases. Paired-end reads were assembled using the CLC *de novo* assembly algorithm, using a kmer length of 63 and a minimum scaffold length of 1 kb. Mate-pair reads were trimmed using NextClip (Leggett *et al.*, 2014) and only reads in class A were used for mapping.

Metagenome binning and data generation was conducted as described previously (Albertsen *et al.*, 2013) using the mmgenome R package and scripts (http://madsalbertsen.github.io/mmgenome/). The genome was manually scaffolded using paired-end and mate-pair connections aided by visualization in Circos (Krzywinski *et al.*, 2009). Gaps were closed using GapFiller (Boetzer and Pirovano, 2012) and manually through inspections of read alignments in CLC Genomics Workbench.

The assembled genome was annotated using both Integrated Microbial Genomes Expert Review (IMG ER; Markowitz *et al.*, 2009) and the MicroScope platform for microbial genome annotation (MaGe; Vallenet *et al.*, 2009). Locus tags are based on MaGe annotations. Comparative analysis of MetaCyc degradation, utilization and assimilation pathways were generated automatically in MaGe and updated manually to remove incorrect automatic assignments. The full genome sequence of *Ca.* N. exaquare G61 has been deposited in GenBank (accession CP017922) and associated annotations are publicly available in both IMG ER (ID 2603880166) and MicroScope (#U7DNPY). Summary data and genome accession numbers for associated enrichment culture bacteria are summarized in Supplementary Table S3.

## Results

### *Ca.* N. exaquare enrichment culture

**Figure 1:**
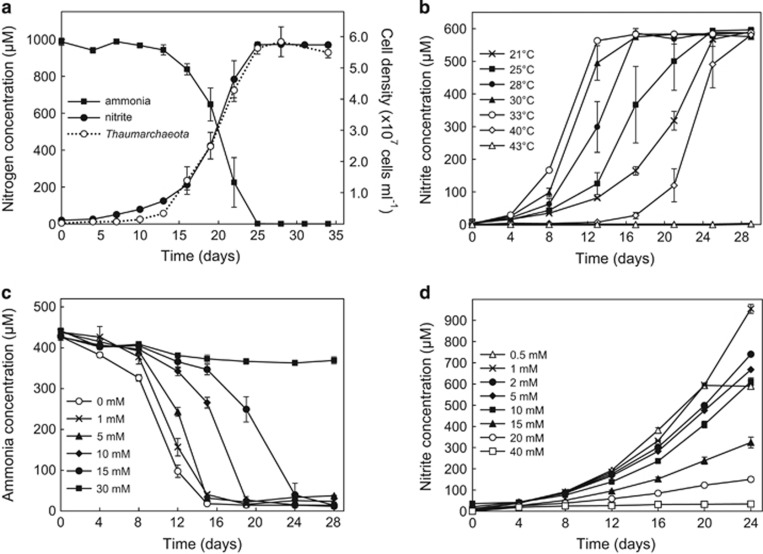
Growth and activity of *Ca.* N. exaquare. (**a**) Growth curve, indicating conversion of 1 mm ammonia to nitrite and corresponding increase in thaumarchaeotal cell numbers. Cell numbers were estimated by thaumarchaeotal 16S rRNA gene copy numbers, halved to account for two 16S rRNA gene copies per genome. (**b**) *Ca.* N. exaquare nitrite production at varying incubation temperatures. (**c**) Depletion of 0.5 mm ammonia by *Ca* N. exaquare at varying initial nitrite concentrations. Starting nitrite concentrations are indicated in the panel legend. For this panel, ammonia depletion is shown due to high background levels of nitrite. (**d**) *Ca.* N. exaquare nitrite production at varying starting ammonia concentrations. Starting ammonia concentrations are indicated in the panel legend. Note that nitrite production plateaus in the 0.5 mm condition due to depletion of supplied ammonia. All incubations were prepared from a 1% inoculum from an actively growing enrichment culture and were performed in the dark, without shaking. Unless otherwise indicated, the incubation temperature was 30 °C. Error bars indicate the standard error of the mean for biological triplicates. Error bars not seen are contained within the symbols.

*Ca.* N. exaquare has been growing in enrichment culture for over three years and, based on quantitative PCR of thaumarchaeotal and bacterial 16S rRNA genes, comprises approximately 99% of cells present. *Ca.* N. exaquare depletes ammonia and produces nitrite at near-stoichiometric levels (Figure 1a), with an associated generation time of 51.7 h, and a cell density of approximately 5.6 × 10^7^ cells ml^−^^1^ (after oxidizing 1 mm NH_4_Cl). *Ca.* N. exaquare grows over a broad temperature range, with optimal ammonia-oxidizing activity at 33 °C and complete inhibition at 43 °C (Figure 1b). Nitrite-free medium results in the fastest ammonia-oxidizing activity by *Ca.* N. exaquare, and activity slows as initial nitrite concentrations increase (Figure 1c). Despite an increased lag time, *Ca.* N. exaquare cells fully oxidized 0.5 mm NH_4_Cl in the presence of up to 15 mm nitrite. Optimal activity was observed at ammonia concentrations of 0.5–1 mm NH_4_Cl, but initiation of ammonia-oxidizing activity persisted up to 20 mm ammonium (Figure 1d). At 15 mM NH_4_Cl, *Ca.* N. exaquare oxidized all supplied ammonia to nitrite, although an incubation of approximately six months was required (data not shown). *Ca*. N. exaquare cells could be revived following short-term and long-term storage at 4 °C and cryopreservation at −80 °C (Supplementary Figures S1A and B).

**Figure 2:**
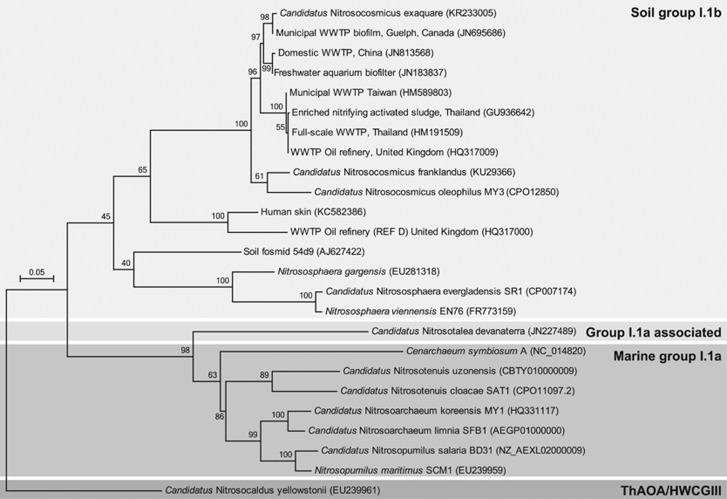
Phylogenetic affiliations of *amoA* gene sequences for *Ca.*N. exaquare and other thaumarchaeotal representatives, inferred using the maximum likelihood method based on the general time reversible model. The tree is drawn to scale, with branch lengths measured as the number of substitutions per site. Bootstrap values are located above branches and are based on 500 replicates. Only bootstrap values >50% are indicated on the tree. The scale bar represents 5% nucleotide divergence.

On the basis of *amoA* (Figure 2) and 16S rRNA (Supplementary Figure S2) gene sequences, *Ca.* N. exaquare belongs to the soil group I.1b *Thaumarchaeota* cluster, specifically in the *Nitrososphaera* sister cluster, and is related to *Ca.* N. franklandus (Lehtovirta-Morley *et al.*, 2016) and *Ca*. N. oleophilus (Jung *et al.*, 2016). In addition, *Ca*. N. exaquare gene sequences are closely related to environmental sequences originating from several municipal and industrial WWTPs. *Ca*. N. exaquare has sequence identities of >99% to *amoA* and 16S rRNA gene sequences obtained from the original Guelph WWTP biofilm.

**Figure 3:**
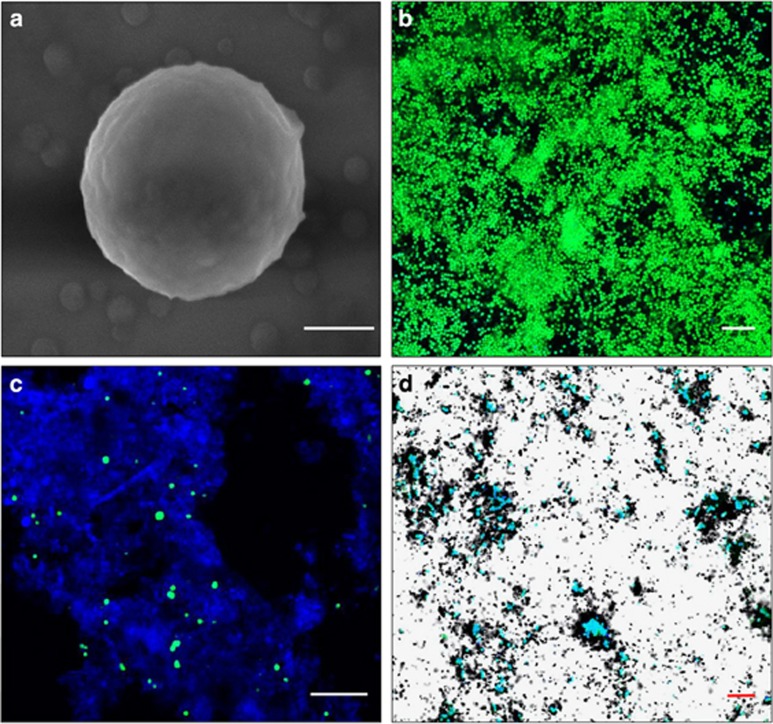
Micrographs of *Ca.*N. exaquare cells. (**a**) Scanning electron micrograph with a 400-nm scale bar (measured cell diameter is 1.3 μm). (**b**) CARD-FISH image of cells in enrichment culture. (**c**) CARD-FISH image of thaumarchaeotal cells in RBC biofilm from the Guelph WWTP. (**d**) CARD-FISH combined with MAR of *Ca.* N. exaquare enrichment culture cells labeled with ^14^C-bicarbonate. CARD-FISH images in **b**–**d** were obtained with probe thaum726 applied together with two competitor probes (compA, compB; see Supplementary Table S2) and FITC-labeled tyramides (green). In these panels, DAPI was applied as a nucleic acid stain (blue) and scale bars are 10 μm.

*Ca.* N. exaquare cells are coccoid and approximately 1.3 μm in diameter (Figures 3a and b). Cells up to ~2 μm in diameter were observed, with large cells often having two discrete regions of nucleic acid (Supplementary Figure S3A). Cells often appeared in pairs or groups, which may be covered in an extracellular matrix (Supplementary Figures S3B and C). *Thaumarchaeota* in RBC biofilm samples were also coccoid with a diameter of 1–2 μm (Figure 3c; Supplementary Figure S3D). *Ca.* N. exaquare cells in enrichment culture assimilated ^14^C-bicarbonate coincident with ammonia-oxidizing activity, as indicated by microautoradiographic signal in association with CARD-FISH labeling of thaumarchaeotal cells (Figure 3d).

**Figure 4:**
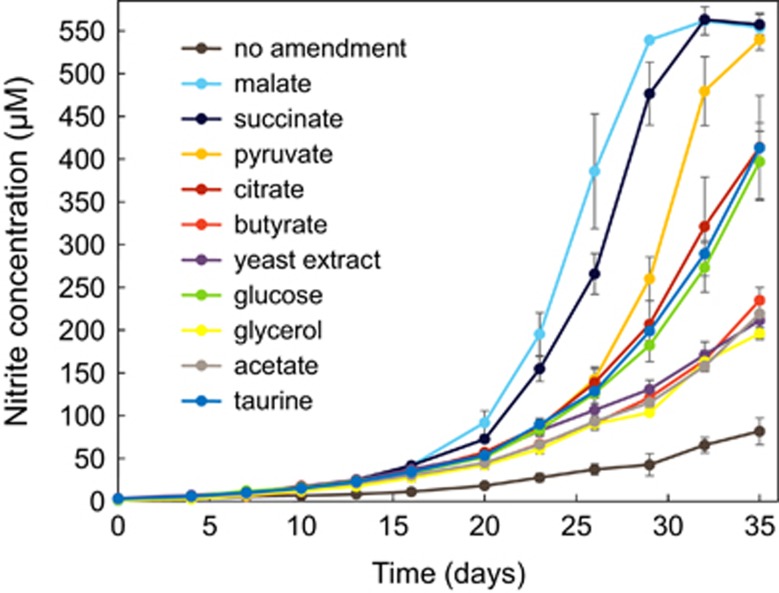
Growth of *Ca.*N. exaquare amended with organic carbon and incubated with 0.5 mM NH_4_Cl at 28 °C. All conditions shown in this figure were performed using the same inoculum and medium batch, and were set-up on the same day. At the time of inoculation, the proportion of *Ca*. N. exaquare in the mixed culture was ~85%, as measured by quantitative PCR for thaumarchaeotal and bacterial 16S rRNA genes. Error bars indicate the standard error of the mean for biological triplicates. Error bars not seen are contained within symbols.

Despite incorporation of inorganic carbon, slowing of growth and activity of *Ca.* N. exaquare cells was observed as the culture became more highly enriched, a pattern reported previously for *N. viennensis* (Tourna *et al.*, 2011). Amendment of the growth medium with various sources of organic carbon stimulated growth of *Ca*. N. exaquare in comparison to a no-organic carbon control (Figure 4). All tested organic carbon compounds stimulated growth, with tricarboxylic acid cycle intermediates malate and succinate providing the most stimulation, followed by pyruvate.

### *Ca.* N. exaquare genome

The genome of *Ca*. N. exaquare was sequenced and assembled using metagenomic binning (Supplementary Figure S4), resulting in one circular contiguous sequence of 2.99 Mbps (Supplementary Figure S5), with very low sequence variation of 478 putative SNPs across the entire genome. The genome has a G+C content of 33.9%, 3162 predicted protein-coding sequences, and a coding density of 77.2%. It encodes 39 tRNA genes, one 5S rRNA gene, and two identical copies each of 16S and 23S rRNA genes (Table 1). Synthesis of several vitamins is encoded, including cobalamin (vitamin B_12_), consistent with a previous study linking marine cobalamin production with *Thaumarchaeota* (Doxey *et al.*, 2015). *Ca*. N. exaquare encodes transporters for amino acids and di/oligopeptides, as well as two sodium-dependent dicarboxylate transporters (A4241_1584, 2720). Several genes encoding enzymes associated with detoxification of reactive oxygen species (ROS) are present in the genome, including catalase (A4241_2297), peroxidase (A4241_2636), superoxide dismutase (A4241_1350), alkyl hydroperoxide reductase/peroxiredoxins and thioredoxins (Table 1). *Ca.* N. exaquare has several genes related to ammonia oxidation, including *amoA*, *amoB*, and three copies of *amoC*, in an arrangement that differs from both AOB and other AOA representatives (Supplementary Figure S6). In addition, *Ca*. N. exaquare encodes urea transporters and urease enzyme subunits and accessory proteins (Table 1), and produces nitrite from urea in enrichment culture (data not shown). Unlike other I.1b *Thaumarchaeota*, *Ca*. N. exaquare does not encode genes associated with chemotaxis or flagellar synthesis (Table 1).

**Figure 5:**
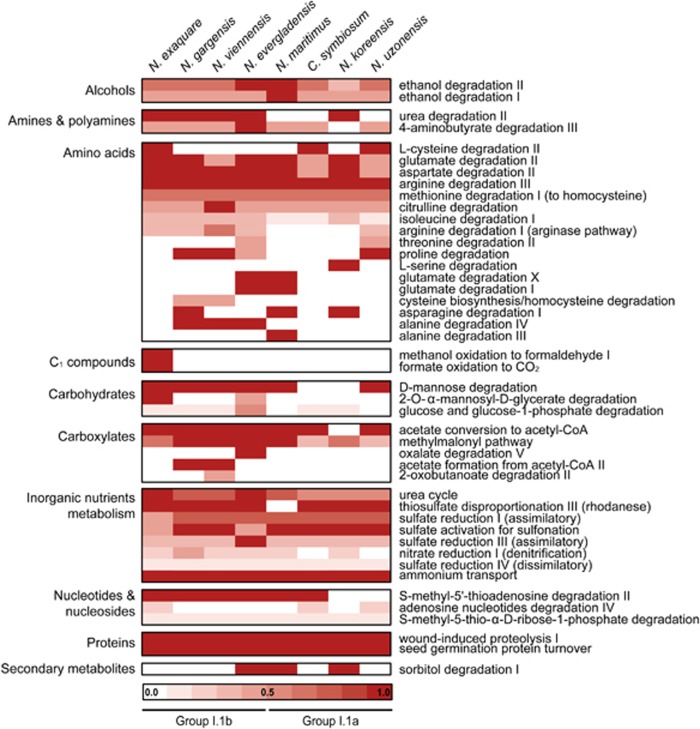
Comparative analysis of MetaCyc degradation pathways of *Ca.*N. exaquare and other selected *Thaumarchaeota*. The heat map was generated using the MicroScope platform and includes all MetaCyc pathways under the category ‘degradation, utilization and assimilation’.

There was little synteny between *Ca.* N. exaquare and *N. maritimus* (Supplementary Figure S7A), *N. gargensis* (Supplementary Figure S7B) or *N. viennensis* (Supplementary Figure S7C). In comparison, *N. gargensis* and *N. viennensis* have several syntenic regions (Supplementary Figure S7D). Average amino acid identities (AAIs) with other AOA were very low, and ranged from 46% with *N. maritimus* to 53% with *N. gargensis* (Supplementary Table S4). MetaCyc degradation pathways encoded by *Ca.* N. exaquare demonstrate metabolic similarity with other AOA representatives, especially group I.1b *Thaumarchaeota* (Figure 5). For example, all AOA representatives share pathways associated with proteolysis and degradation of amino acids. Few pathways involved in carbohydrate metabolism were identified, with the only unique MetaCyc pathway involving degradation of 2-*O*-α-mannosyl-d-glycerate. Automated annotation of the *Ca.* N. exaquare genome also suggested pathways associated with one-carbon (C_1_) compound utilization, including methanol oxidation to formaldehyde, and formate oxidation to CO_2_ (Figure 5). In addition, *Ca*. N. exaquare encodes two copies of S-(hydroxymethyl)glutathione (A4241_3046, 3091), which oxidizes formaldehyde in the presence of glutathione.

### Guelph WWTP biofilm

Inhibitors used for biofilm activity assays were first tested on *Ca*. N. exaquare and *N. europaea*. Oxidation of 0.5 mm NH_4_Cl by *Ca*. N. exaquare was only inhibited by relatively high concentrations of ATU (⩾100 μm; Supplementary Figure S8A) and was not inhibited by octyne at any tested concentration (up to 30 μm; Supplementary Figure S8B). Conversely, PTIO was strongly inhibitory at 30 μm, with total inhibition observed at 100 μm (Supplementary Figure S8C), consistent with previous observations for this organism (Sauder *et al.*, 2016). For *N. europaea*, 10 μm ATU and 8 μm octyne completely inhibited the oxidation of 0.5 mm NH_4_Cl, whereas inhibition was not observed by 200 or 400 μm PTIO (Supplementary Figure S9).

**Figure 6:**
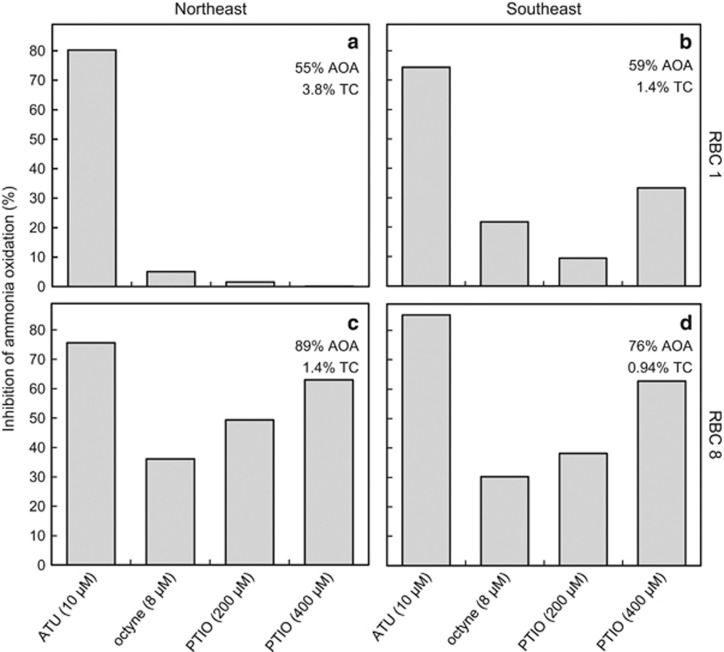
Ammonia oxidation by RBC biofilm in the presence of nitrification inhibitors. Biofilm samples were incubated in wastewater supplemented with 1 mm NH_4_Cl. Percent inhibition of ammonia oxidation is compared with a no-inhibitor control after 42 h of incubation. See Supplementary Figure S10 at ammonia concentrations for all time points. % AOA depicts the proportion of AOA *amoA* gene of *amoA* genes of AOA and AOB detected by qPCR in these samples. %TC represents the relative abundance of AOA to the total community (TC), as measured by qPCR of total bacterial plus thaumarchaeal 16S rRNA genes (Supplementary Table S5).

Biofilm samples obtained from Guelph RBCs (December 2015) oxidized 1 mm NH_4_Cl within 2–4 days (Supplementary Figure S10). Acetylene control flasks showed no ammonia depletion over the incubation period. In fact, an increase in ammonia concentration was observed, likely due to mineralization. Compared with no-inhibitor controls, ATU inhibited between 75% and 85% of ammonia oxidation after 42 h of incubation (Figure 6). Octyne amendment resulted in less inhibition of ammonia oxidation (5–36%). Addition of 200 μm PTIO resulted in little inhibition in RBC 1 biofilm (2–9%) with more inhibition in RBC 8 samples (38–49%). With 400 μm PTIO, patterns were similar, but inhibition was stronger in RBC 8 biofilm samples (63%). In April 2015, similar activity experiments were performed with Guelph RBC biofilm and inhibitors, and demonstrated that ammonia-oxidizing activity in all tested RBCs was partially inhibited by the addition of 200 μm PTIO and strongly inhibited by 10 μm ATU (Supplementary Figure S11).

In RBC 1 and 8 biofilm samples, *Thaumarchaeota* comprised 55–60% and 76–89% of the total putative ammonia oxidizers, respectively (Figure 6; Supplementary Table S5). Although the proportion of thaumarchaeota was higher in RBC 8, the absolute abundance of AOA-associated genes per ng genomic DNA was approximately 3-fold higher in genomic DNA from RBC 1 biofilm. The absolute abundance of AOB was also higher in RBC 1 biofilm compared with the corresponding RBC 8 samples (Supplementary Table S5). Overall, AOA contributed between 0.9% and 3.8% of the total 16S rRNA genes measured in RBC biofilm samples (Figure 6). Quantitative FISH performed for SE biofilm samples (at an earlier time point) provided similar results, with AOA comprising 4.2% (RBC 1) and 2.4% (RBC 8) of the total DAPI stained biomass. In comparison, AOB comprised 1% (RBC 1) and 0.6% (RBC 8) of the total biomass.

**Figure 7:**
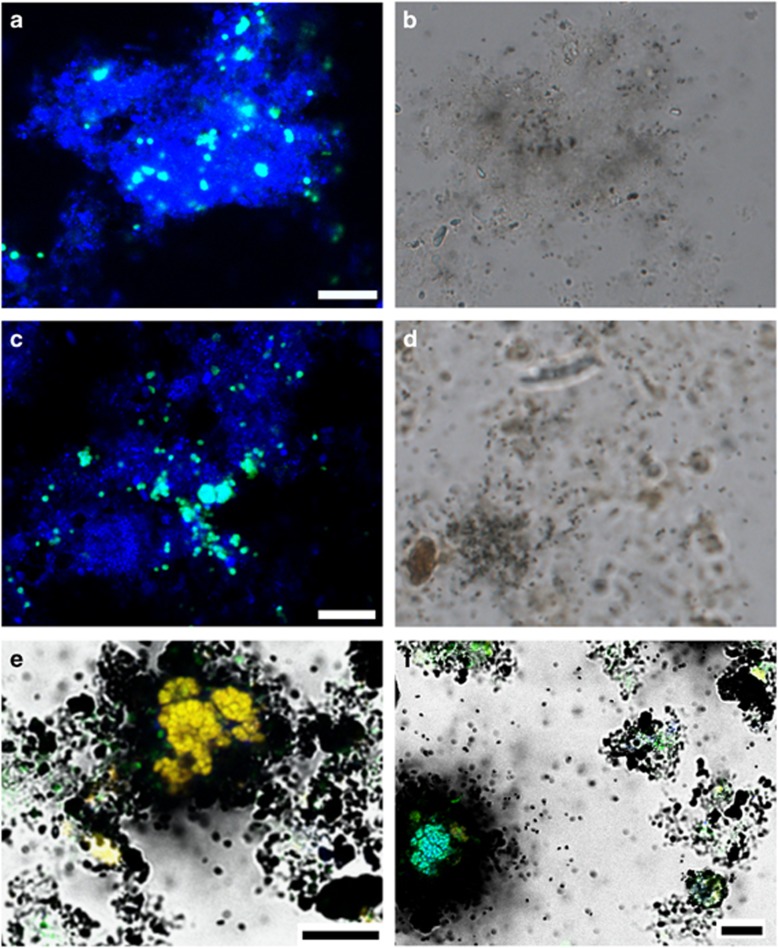
CARD-FISH-MAR for thaumarchaeotes in RBC 1 (**a** and **b**) and RBC 8 (**c** and **d**) biofilm. FISH-MAR for AOB (orange/yellow) and *Nitrospira* (blue) (**e** and **f**; both RBC 8). Green signals in **e** and **f** originate from the EUB338 probe mix targeting most bacteria. Microautoradiographic images were taken with the CCD black/white camera integrated in the CLSM (**e** and **f**) or with an external CCD color camera (DFC 450, Leica Microsystem, Wetzlar, Germany; **b** and **d**). All scale bars represent 10 μm.

In CARD-FISH-MAR experiments, the thaumarchaeotes in SE RBC 1 and RBC 8 did not show incorporation of ^14^C-labeled bicarbonate in the presence of 0.5 mm ammonia after incubation for 6 or 20 h, using MAR exposure times of 9, 12 and 15 days (Figure 7). MAR results were negative for many thaumarchaeotal cells, but no statement could be made for some cells located in proximity to AOB or *Nitrospira*. In contrast, positive MAR signals were obtained for many AOB and *Nitrospira* microcolonies in both RBC 1 and 8 (Figure 7). In RBC 1, following 6 h and 20 h of incubation, 59% and 71% of AOB colonies, and 14% and 36% of *Nitrospira* colonies, were MAR-positive, respectively. In RBC 8, following 6 and 20 h of incubation, 46% and 86% of AOB colonies, and 8% and 16% of the *Nitrospira* microcolonies were MAR-positive, respectively. In no-ammonia control experiments, no MAR-positive AOB nor NOB microcolonies were detected in RBC 8. In RBC 1, up to 10% of the AOB and 7% of the *Nitrospira* microcolonies showed a positive MAR signal, which was most likely the result of autotrophy associated with ammonia mineralization. Control experiments with dead biofilm samples resulted in no detectable MAR-positive cells, excluding chemographic effects.

## Discussion

Little is known about the metabolism and activity of *Thaumarchaeota* detected in WWTPs, despite the importance of these environments to human and environmental health. Here, we report the cultivation of a *Thaumarchaeota* representative originating from a municipal WWTP, which oxidizes ammonia, fixes inorganic carbon, and possesses a genomic repertoire consistent with chemolithoautotrophy. The genus name is based on the related organisms *Ca*. N. franklandus (Lehtovirta-Morley *et al.*, 2016) and *Ca.* N. oleophilus (Jung *et al.*, 2016), and the species name ‘*exaquare*’ (latin for ‘water running out’ or ‘sewage’) reflects its wastewater origin.

*Ca*. N. exaquare produces nitrite from ammonia at near-stoichiometric values (Figure 1a), and thaumarchaeotal cell numbers follow nitrite production closely, providing evidence that energy for cell growth is derived from the oxidation of ammonia to nitrite. The 51.7 h generation time of *Ca*. N. exaquare is similar to that originally reported for *N. viennensis* (46 h), although a shorter generation time of 27.5 h was later reported (Stieglmeier *et al.*, 2014). *Ca.* N. exaquare is mesophilic, with optimal growth observed at 33 °C (Figure 1b). No growth was observed for *Ca.* N. exaquare above 40 °C, in contrast to *N. gargensis*, which grows optimally at 46 °C (Hatzenpichler *et al.*, 2008) and *N. viennensis*, which can tolerate temperatures of at least 47 °C (Tourna *et al.*, 2011).

*Ca.* N. exaquare can withstand relatively high concentrations of both ammonia and nitrite (Figures 1c and d). Ammonia oxidation proceeded in the presence of up to 15 mm NaNO_2_, with complete inhibition observed at 30 mm. *N. viennensis* oxidizes ammonia with little inhibition at 10 mm NaNO_2_, but ammonia oxidation ceased if ~3.5 mm nitrification-derived nitrite accumulated (Tourna *et al.*, 2011). In contrast, *Ca.* N. exaquare fully oxidizes at least 15 mm NH_4_Cl, indicating that it may be better able to tolerate nitrite or other ammonia oxidation intermediates. Initiation of ammonia oxidation can be achieved with ammonia concentrations of up to 20 mm, with complete inhibition not observed until 30 mm (Figure 1d). For the growth conditions used (that is, pH 8, 30 °C), 20 mm NH_4_Cl is equivalent to 1.49 mm un-ionized ammonia (NH_3_). For comparison, reported inhibitory concentrations of un-ionized ammonia are only 18–27 μm, <9 μm and 0.51–0.75 μm for *N. maritimus*, *Ca.* N. devanaterra and *N. viennensis*, respectively (see Hatzenpichler, 2012 for a review). High tolerance to ammonia is perhaps unsurprising given that *Ca.* N. exaquare originates from a municipal WWTP, where ammonia concentrations would be higher than in most naturally occurring soil or aquatic environments. Niche partitioning occurs between AOA and AOB based on ammonia availability (Erguder *et al.*, 2009; Jia and Conrad, 2009; Martens-Habbena *et al.*, 2009; Schleper, 2010; Verhamme *et al.*, 2011; Sauder *et al.*, 2012), but given the high diversity of AOA and their global distribution across diverse environments, it is also possible that niche partitioning based on ammonia concentrations also occurs within the *Thaumarchaeota.* Similarly, nitrite has been suggested as a major driver of niche partitioning for nitrite-oxidizers from the genus *Nitrospira* (Maixner *et al.*, 2006).

*Ca.* N. exaquare is the first reported representative of the *Nitrososphaera* sister cluster originating from a WWTP. Both *amoA* (Figure 2) and 16S rRNA (Supplementary Figure S2) gene sequences clustered with other *Thaumarchaeota* from WWTPs, both industrial and municipal, as well as other waste-related environments such as landfills and landfill-contaminated soils. Most detected thaumarchaeotal sequences from WWTPs affiliate with the I.1b soil group, often in the *Nitrososphaera* sister cluster (Mussmann *et al.*, 2011; Sauder *et al.*, 2012; Gao *et al.*, 2013; Limpiyakorn *et al.*, 2013). These engineered environments represent comparatively nutrient-rich habitats, characterized by relatively high levels of organic carbon and ammonia. Combined with the observed high tolerance to ammonia and nitrite, this clustering could reflect an adaptation of *Ca.* N. exaquare-like *Thaumarchaeota* to high nutrient environments.

With cell sizes up to 2 μm in diameter, *Ca.* N. exaquare is the largest reported member of the *Thaumarchaeota*. Most observed cells were ~1.3 μm (Figure 3a), which is substantially larger than group I.1a *Thaumarchaeota* (for example, *N. maritimus* cells are 0.2 μm × 0.7 μm; Könneke *et al.*, 2005). Group I.1b *Thaumarchaeota* are larger, including *N. gargensis* and *N. viennensis*, which have cell diameters of ~0.9 μm and 0.5–0.8 μm, respectively (Hatzenpichler *et al.*, 2008; Tourna *et al.*, 2011). Although coccoid morphologies have been reported for all group I.1b *Thaumarchaoeta*, *Ca*. N. exaquare cells appear smoothly spherical (Figure 2a), whereas *N. viennensis* cells are irregular, with concave areas that appear collapsed into the cell (Tourna *et al.*, 2011). The cell size and morphology of *Ca.* N. exaquare closely resemble thaumarchaeotal cells previously detected by CARD-FISH in wastewater sludge samples from industrial WWTPs (Mussmann *et al.*, 2011).

*Ca.* N. exaquare incorporated bicarbonate into biomass in association with ammonia oxidation (Figure 3d) and encodes the 3-hydroxypropionate/4-hydroxybutyrate (3HP/4HB) carbon fixation pathway (Table 1), which is used by all known *Thaumarchaeota* (Berg *et al.*, 2007; Berg, 2011). Moreover, it has grown in enrichment culture for several years without any externally supplied organic carbon. These data indicate that *Ca.* N. exaquare combines ammonia oxidation with autotrophic carbon fixation, as expected for a classical ammonia-oxidizing microorganism. Despite this, *Ca.* N. exaquare is strongly stimulated by the addition of organic carbon (Figure 4), which may indicate a mixotrophic metabolism, or an indirect benefit. A variety of organic carbon sources accelerated ammonia-oxidizing activity by *Ca.* N. exaquare, with malate and succinate resulting in the highest level of stimulation (Figure 4). *Ca.* N. exaquare may be able to incorporate these metabolic intermediates directly into its tricarboxylic acid cycle, which could provide reducing power or precursors for biomolecule synthesis. However, given the wide variety of stimulatory compounds (for example, glycerol, yeast extract, butyrate), it is likely that not all organic carbon sources directly stimulate growth, but instead provide indirect benefits via remaining heterotrophic bacteria. A mixotrophic lifestyle would be consistent with previous environmental observations. For example, marine archaea assimilate amino acids (Ouverney and Fuhrman, 2000), and radiocarbon analyses of the membrane lipids of pelagic marine *Thaumarchaeota* indicate that communities are composed of combination of autotrophs and heterotrophs, or a single mixotrophic population (Ingalls *et al.*, 2006). In addition, *N. viennensis* and marine thaumarchaeotal strains require pyruvate or α-ketoglutaric acid for optimal growth (Tourna *et al.*, 2011; Qin *et al.*, 2014), although the mechanism of action of these compounds is detoxification of ROS (Kim *et al.*, 2016). Although ROS detoxification was not demonstrated with succinate, it is possible that this compound stimulates growth of heterotrophs that in turn detoxify ROS and thereby encourage growth of AOA. However, *Ca*. N. exaquare encodes a variety of genes that may confer protection from ROS: in addition to several genes shared among many *Thaumarchaeota* (for example, superoxide dismutase, alkyl hydroperoxide reductase), *Ca*. N. exaquare encodes a peroxidase, which is unique among sequenced thaumarchaeotal genomes, and a manganese-dependent catalase, which is also present in *Ca*. N. evergladensis (Table 1).

The genome of *Ca.* N. exaquare encodes two gene copies of a sodium-dependent dicarboxylate transporter (SdcS; Table 1), which transports succinate, malate and fumarate (Hall and Pajor, 2005, 2007). SdcS-type dicarboxylate transporters are also encoded in the genomes of *Ca*. N. evergladensis (Zhalnina *et al.*, 2014) and *Ca.* N. uzonensis (Lebedeva *et al.*, 2013). Expression of this transporter could provide an explanation for the observed stimulation of *Ca*. N. exaquare by succinate and malate (Figure 4). In addition to being a tricarboxylic acid cycle intermediate, succinate is a central compound in the 3HB/4HP cycle (Berg, 2011) and could feed directly into this carbon fixation pathway. Labeling studies with *Metallosphaera medulla*, which also uses the 3HP/4HP cycle (Berg *et al.*, 2007), demonstrated that the majority of anabolic precursors are derived from succinate (Estelmann *et al.*, 2011). Given the presence of this transporter and the strong stimulatory effects of succinate and malate, C_4_ compounds may have an important role in supplementing *Ca.* N. exaquare metabolism.

At 2.99 Mbps, *Ca*. N. exaquare encodes the largest reported AOA genome, and shares several features with Group I.1b soil *Thaumarchaeota* (Table 1). Interestingly, *Ca.* N. exaquare has a G+C content (33.9%) that is lower than other Group I.1b *Thaumarchaeota* (~50%), but comparable to group I.1a *Thaumarchaeota*. The genome encodes all key components for ammonia oxidation and bicarbonate fixation pathways, supporting its role as a chemolithoautotrophic ammonia oxidizer. In addition, *Ca.* N. exaquare has a similar metabolic profile to other AOA (Figure 5), with few genes associated with carbohydrate catabolism. An encoded pathway for degradation of mannosylglycerate was identified as unique among thaumarchaeotal genomes, but most likely relates to osmostic regulation, which has been suggested previously (Spang *et al.*, 2012; Zhalnina *et al.*, 2012). *Ca.* N. exaquare also encodes genes associated with C_1_ metabolism, including formate dehydrogenase and glutathione-dependent formaldehyde dehydrogenase. Several autotrophic NOB can oxidize or assimilate formate (Malavolta *et al.*, 1962; Van Gool and Laudelout, 1966; Gruber-Dorninger *et al.*, 2015; Koch *et al.*, 2015). However, these encoded enzymes could be used for detoxification, and further work is necessary to assess whether C_1_ substrates could supplement autotrophic metabolism.

AOA have been detected and quantified in several WWTPs, but only one study has assessed the relative contributions of ammonia-oxidizing prokaryotes to ammonia oxidation in WWTPs. Mussman *et al.* (2011) detected *Thaumarchaeota* in industrial WWTPs treating oil refinery waste but found no evidence for bicarbonate fixation, despite active expression of *amoA* genes. These *Thaumarchaeota* are phylogenetically related (Figure 2) and morphologically similar to *Ca.* N. exaquare, which oxidizes ammonia, assimilates bicarbonate and encodes a genome supporting chemolithoautotrophic metabolism. However, ammonia monooxygenase substrate promiscuity has been reported (Pester *et al.*, 2011), and different growth conditions elicit different physiological responses, so the role of *Ca.* N. exaquare *in situ* is likely more complex in natural environments.

Incubations of Guelph WWTP RBC biofilm with differential inhibitors indicated that ATU was highly inhibitory, octyne had little effect and PTIO was partially inhibitory. More inhibition by PTIO was observed in RBC 8 biofilms compared with RBC 1 of the same treatment train, suggesting that a larger proportion of the ammonia-oxidizing activity results from AOA. This is supported by qPCR data demonstrating that thaumarchaeotes comprise a higher proportion of the putative ammonia-oxidizing prokaryotes in RBC 8 compared with RBC 1 (Figure 6; Supplementary Table S5). Octyne and ATU are specific inhibitors of AOB (Hatzenpichler *et al.*, 2008; Shen *et al.*, 2013; Taylor *et al.*, 2013) and results obtained from these compounds should ideally be similar, but were inconsistent in this study. Several advantages have been reported for octyne (Taylor *et al.*, 2013), but it has not been used previously with samples from a WWTP environment, and may have been degraded by biofilm microorganisms. Two PTIO concentrations were included because lower concentrations may be insufficient in environmental samples due to production of nitric oxide from non-nitrification processes (for example, denitrification), but higher concentrations might result in inhibition of some AOB. For example, PTIO concentrations of 400 μm are partially inhibitory to *N. multiformis* (Shen *et al.*, 2013), although not to *N. europaea* (Supplementary Figure S9).

Although questions remain regarding the efficacy of octyne at inhibiting AOB in this biofilm, and whether AOB were also inhibited using 400 μm PTIO, the observed inhibition of ammonia-oxidizing activity by 200 μm PTIO suggests that *Thaumarchaeota* contribute to ammonia-oxidizing activity of the biofilm. The previously reported relationship between ammonia concentration and thaumarchaeotal abundance in this biofilm (Sauder *et al.*, 2012) supports the role of *Ca*. N. exaquare-like AOA as ammonia oxidizers *in situ*. Although this work only considers AOA and AOB, completely nitrifying *Nitrospira* organisms (that is, comammox bacteria; van Kessel *et al.*, 2015; Daims *et al.*, 2015), with unknown sensitivities to these inhibitors, could be contributing to nitrification activity in the biofilm.

The CARD-MAR-FISH data from the RBC biofilm samples indicated that when supplied with ammonia, both AOB and *Nitrospira* are MAR-positive, whereas there was no evidence for bicarbonate fixation by *Thaumarchaeota* (Figure 7). This suggests that the detected *Thaumarchaeota* cells were either predominantly relying on an alternative metabolism, or that they were oxidizing ammonia for energy but assimilating a carbon source other than bicarbonate. Similarly, related *Thaumarchaeota* in another WWTP did not assimilate inorganic carbon in the presence of ammonia (Mussmann *et al.*, 2011). Most observed AOB showed strong MAR signals, which is consistent with the strongly inhibitory effect of ATU. Positive MAR signals were observed for some *Nitrospira* microcolonies (Figure 7), which could have arisen from either nitrite-oxidizing or comammox activity.

*Ca.* N. exaquare is the first group I.1b *Thaumarchaeota* representative cultivated from a WWTP, and clusters phylogenetically with AOA originating from wastewater environments. The laboratory activity and genetic complement of *Ca.* N. exaquare suggest that it is a classic ammonia-oxidizing microorganism, which may be stimulated by organic carbon. In the wastewater biofilm from which it originates, both qPCR and qFISH indicate that *Thaumarchaeota* consistently outnumber AOB. However, the metabolic role played *in situ* by *Ca*. N. exaquare appears to be more complex than strictly chemolithoautotrophic nitrification, and it is possible that the relatively high abundance of *Thaumarchaeota* could be explained by growth on other substrates present in the biofilm. Further work is needed to elucidate the contributions of *Ca*. N. exaquare-like *Thaumarchaeota* to ammonia oxidation in this system and to assess the *in situ* potential for autotrophic or mixotrophic metabolisms.

## Figures and Tables

**Table 1:** Genome features of *Ca.* N. exaquare G61 and other selected members of the *Thaumarchaeota*

*Genome features*	*Nitrosocosmicus exaquare* G61	*Nitrososphaera gargensis* Ga9-2^a^	*Nitrosophaera viennensis* EN76^b^	*Nitrososphaera evergladensis* SR1^c^	*Nitrosopumilus maritimus* SCM1^d^
Cluster	I.1b	I.1b	I.1b	I.1b	I.1a
Genome size (Mb)	2.99	2.83	2.53	2.95	1.6
Contigs	1	1	1	1	1
GC (%)	33.9	48.4	52.7	50.14	34.2
Total genes	3206	3609	3167	3548	1847
Protein-coding genes	3162	3566	3027	3505	1796
Coding density (%)	77.2	81.5	87.3	83.6	90.8
16-23S rRNA	2	1	1	1	1
5S rRNA	1	1	1	1	1
tRNA	39	40	39	39	44
CRISPR loci	2	1	2	1	−
Cell division	FtsZ, Cdv	FtsZ, Cdv	FtsZ, Cdv	FtsZ, Cdv	FtsZ, Cdv
Motility/chemotaxis	−	+	+	+	−
Carbon fixation	3HP/4HB	3HP/4HB	3HP/4HB	3HP/4HB	3HP/4HB
Ammonium transporters	1	3	3	3	2
NirK	1	1	1	1	1
Urease and urea transport	+	+	+	+	−
Cyanate lyase	−	+	−	−	−
Coenzyme F_420_	+	+	+	+	+
Vitamin B_12_ (cobalamin) synthesis	+	+	+	+	+
Polyhydroxyalkanoate synthesis	+	+	+	+	−
Dicarboxylate transporter (SdcS)	2	−	−	−	1
Amino acid transporters	+	+	+	+	−
Dipeptide/oligopeptide transporters	+	+	+	+	+
Catalase (Mn-containing)	1	1^e^	0	1	0
Peroxidase	1	0	0	0	0
Superoxide dismutase	1	1	1	1	2
Alkyl hydroperoxide reductases	+	+	+	+	+
Thioredoxins	+	+	+	+	+

a Spang *et al.* (2012).

b Stieglmeier *et al.* (2014).

c Zhalnina *et al.* (2014).

d Walker *et al.* (2010).

e Truncated gene.
