# Correction: Cultivation and characterization of Candidatus Nitrosocosmicus exaquare, an ammonia-oxidizing archaeon from a municipal wastewater treatment system

**DOI:** 10.1038/s41396-020-0714-3

**Published:** 2020-07-10

**Authors:** Laura A. Sauder, Mads Albertsen, Katja Engel, Jasmin Schwarz, Per H. Nielsen, Michael Wagner, Josh D. Neufeld

**Affiliations:** 1 Department of Biology, University of Waterloo, Waterloo, ON Canada; 2 Center for Microbial Communities, Department of Chemistry and Bioscience, Aalborg University, Aalborg, Denmark; 3 Department of Microbiology and Ecosystem Science, Division of Microbial Ecology, Research Network ‘Chemistry meets Microbiology’, University of Vienna, Vienna, Austria

Correction to: *The ISME Journal*

10.1038/ismej.2016.192

After publication of this article, the authors discovered that the species name “exaquare” is inconsistent with Latin naming conventions. As a result, they hereby correct the proposed species name of this ammonia-oxidizing archaea representative to Candidatus Nitrosocosmicus hydrocola (hy.dro’co.la. Gr. n. hydor, water; L. suff. -cola, inhabitant, dweller; N.L. masc. adj. hydrocola, an inhabitant of water, referring to its growth within a wastewater treatment system).

The authors acknowledge Latin assistance from Professor Bernhard Schink.

