# Supplementary material for: Cultivation and characterization of Candidatus Nitrosocosmicus exaquare, an ammonia-oxidizing archaeon from a municipal wastewater treatment system
Source: ISME J. 2017 Feb 14;11(5):1142–57. doi: 10.1038/ismej.2016.192 (PMC5398378; doi:10.1038/ismej.2016.192)
Supplement: Supplementary Information [file ismej2016192x1.docx]

**Supplementary Information**

**Cultivation and characterization of *Candidatus* Nitrosocosmicus exaquare, an ammonia-oxidizing archaeon from a municipal wastewater treatment system**

Laura A. Sauder^1^, Mads Albertsen^2^, Katja Engel^1^, Jasmin Schwarz^3^, Per H. Nielsen^2^, Michael Wagner^3^, Josh D. Neufeld^1*^

^1^Department of Biology, University of Waterloo, Waterloo, Ontario, Canada

^2^Center for Microbial Communities, Department of Chemistry and Bioscience, Aalborg University, Aalborg, Denmark

^3^Department of Microbiology and Ecosystem Science, Division of Microbial Ecology, Research Network “Chemistry meets Microbiology”, University of Vienna, Vienna, Austria

*Corresponding author: Department of Biology, University of Waterloo, 200 University Avenue West, Waterloo, Ontario, N2L 3G1, Canada. Tel. +1 519-888-4567; Fax +1 519-746-0614. E-mail: jneufeld@uwaterloo.ca.

Running title: Cultivation and characterization of *Candidatus* Nitrosocosmicus exaquare

Keywords: nitrification, *Thaumarchaeota*, ammonia-oxidizing archaea, nitrification, wastewater treatment

**SI materials and methods**

*AOA cultivation*

Cultures were originally set up using freshwater media (FWM) as described previously (Tourna *et al.*, 2011). In the early stages of the enrichment culture, growth media was supplemented with kanamycin (50 µg/ml) and streptomycin (100 µg/ml) to inhibit growth of Gram-negative bacteria (especially AOB) and ampicillin (50 µg/ml) to inhibit growth of Gram-positive bacteria. Stock cultures were monitored for ammonia and nitrite concentrations at least once weekly, and were transferred to fresh medium or fed additional substrate when greater than three quarters of the supplied ammonia was depleted. After approximately one year, stock cultures were transferred to a calcium carbonate media, as described in the main manuscript. Unless otherwise stated, all described experiments were performed using the calcium carbonate based media

*Cryopreservation and 4°C storage of* Ca. *N. exaquare*

To test viability of *Ca.* N. exaquare enrichment culture at 4°C, duplicate cultures were stored refrigerated for 0, 1, 3 and 6 months. For all time points (including 0 months), the culture used for inoculation was cooled to 4ᵒC prior to inoculation to control for the effects of cooling on the cells. After the indicated storage time, cultures were inoculated in fresh medium containing 0.5 mM NH_4_Cl (10% transfer) and incubated in the dark at 28°C, without shaking. Samples for water chemistry were stored at -20°C until processing.

Cell were cryopreserved as described previously (Herbold CW, Lebedeva E, Palatinszky M, 2016) with minor modifications. Briefly, cells were frozen in 1 ml of actively growing *Ca.* N. exaquare enrichment culture was placed in a 2-ml screw cap cryo-tube and 1 volume of cryoprotectant was added. Cryoprotectants used were 14% DMSO (7% final concentration) or 70% glycerol (35% final concentration). In both cases, cryoprotectants were prepared in media salts and 0.22-µm filter sterilized. Tubes were inverted several times to mix and were then placed in dry ice. When tube contents were completely frozen, they were moved to -80°C for storage. Cells were resuscitated after two weeks to assess the impact of freezing and cryopreservation treatment, and after one year to assess the impact of long term storage of cells. To resuscitate cells, tubes were thawed at room temperature for 10 minutes, and pelleted during centrifugation at 15,000 x g for 5 minutes. Supernatant was aspirated and discarded, and pellets were washed two times to remove residual DMSO or glycerol. To wash, 1 ml fresh medium was added to tubes, which were then centrifuged as above, and supernatant was removed. After washing, pellets were suspended in 2 ml growth medium, which was then used for inoculum into fresh media containing 0.5 mM NH_4_Cl (5% transfer). Flasks were then incubated in the dark at 28°C and without shaking. Samples for water chemistry analysis were collected at 2 day intervals, and stored at -20°C until processing. No-freeze control flasks were included that were treated with cryoprotectant as outlined, but were not frozen and were inoculated directly into fresh medium. All conditions were performed using biological duplicates.

*quantitative PCR*

qPCR amplifications (10 µl total volume) contained 5 µl iQ SYBR Green Supermix (Bio-Rad), 3 pmol of each primer, 2.5 µg of bovine serum albumin, and 0.1–5 ng of genomic DNA. For thaumarchaeotal and bacterial 16S rRNA genes, the qPCR conditions were 98°C for 2 min, followed by 35 cycles of 98°C for 30 s, 55°C for 30 s, and 72°C for 30 s. Melt curves were performed from 65°C to 95°C, with incremental temperature increases of 0.5°C. For *amoA* genes, conditions were the same, except the elongation time was 1 minute. Standard curves were constructed using 10-fold serial dilutions of template DNA of known concentration. For all genes, template DNA consisted of PCR amplicons generated from the same primer pair used for qPCR. For thaumarchaeotal 16S rRNA gene amplicons, the template source was genomic DNA extracted from the *Ca.* N. exaquare enrichment culture. For AOB *amoA*, the template source was genomic DNA extracted from Guelph WWTP biofilm, and for bacterial 16S rRNA genes, the template source was genomic DNA from *Escherichia coli*.

Double Labeling*of Oligonucleotide Probes (DOPE)-FISH*

Biofilm samples were fixed with 4% PFA and dehydrated in ethanol as described in the main manuscript. Samples were hybridized with double labeled probes, including thaum726 double labelled with fluorescein and applied with unlabelled competitor probes (Supplementary Table S2), and EUB mix (Supplementary Table S2), double labelled with Cy3. Probes were hybridized in 25% hybridization buffer at 46ᵒC for 90 minutes in a humid chamber containing the same formamide concentration, then washed for 15 minutes at 48ᵒC in corresponding wash buffer (0.15 mM NaCl, 20 mM tris, 5 mM EDTA), rinsed with ice cold distilled water and dried using compressed air.

*Genome assembly details*

Metagenome binning and data generation was conducted as described previously (Albertsen *et al.*, 2013) using the mmgenome R package and scripts (<http://madsalbertsen.github.io/mmgenome/>). Coverage profiles were generated by mapping reads to the metagenome scaffolds using the CLC Map Reads to Reference algorithm with a minimum similarity of 95% over 90% of the read length. Open reading frames were predicted in the assembled scaffolds using the metagenome version of Prodigal (Hyatt *et al.*, 2010). A set of 107 HMMs of essential single-copy genes (Dupont *et al.*, 2012) were searched against the predicted open reading frames using HMMER3 (http://hmmer.janelia.org/) with default settings, except the trusted cutoff was used (-cut_tc). The identified proteins were classified taxonomically using BLASTP against the RefSeq protein database (version 52) with a maximum e-value cutoff of 1e-5. MEGAN (Huson *et al.*, 2011) was used to extract class level taxonomic assignments from the BLAST .xml output file. The script network.pl was used to extract paired-end and mate-pair read connections between scaffolds using a SAM file of the read mappings to the metagenome. Metagenome binning was conducted using the mmgenome package in R and can be fully recreated using the R markdown file available at <http://madsalbertsen.github.io/mmgenome/>.

*Average amino acid identity and genome synteny plots*

The average amino acid identity (AAI) values of protein coding genes were calculated using online tools (<http://enve-omics.ce.gatech.edu/ani/>), implementing methods described previously (Goris *et al.*, 2007; Konstantinidis and Tiedje, 2005; Rodriguez and Konstantinidis, 2016). Initially, average nucleotide identities (ANIs) were calculated, but all values were below thresholds considered appropriate for this analysis (75-80%; Rodriguez and Konstantinidis, 2014; 2016). Protein coding sequences used for AAI calculations were based on MicroScope/MaGe protein-coding gene predictions. AAIs were calculated based on reciprocal best hits (blastp) with a minimum alignment of 50% and a minimum identity of 20%.

Full genome alignments and dot plots between *Ca*. N. aquariensis and other AOA genomes were generated using PROmer (Kurtz *et al.*, 2004). Six-frame amino acid translations of input DNA sequences were used to identify matches, and a match length of six amino acids was used. Blue points on the dot plot indicate matches found on parallel strands and red points indicate matches on antiparallel strands.

*Incubation of* Ca*. N. exaquare with PTIO, ATU and octyne*

To assess the sensitivity of the ammonia-oxidizing activity of *Ca.* N exaquare to commonly used differential nitrification inhibitors, actively growing cells were incubated with varying concentrations of PTIO, ATU and octyne (Fig. S8). Incubations were prepared using a 10% inoculum of *Ca.* N. exaquare cells into fresh medium containing 0.5 mM NH_4_Cl. Incubations containing octyne and the associated control were performed in 125-ml serum bottles containing silicone stoppers, and incubations containing PTIO or ATU (and associated controls) were performed in 100-ml Schott bottles with screw caps. Triplicate bottles were incubated in the dark at 28°C without shaking. Samples for water chemistry analyses were stored at -20°C until analysis.

*Incubation of* Nitrosomonas europaea *with PTIO, ATU and octyne*

*N. europaea* was used as a positive control to ensure that inhibitor assays functioned as expected on an AOB representative. For these experiments, *N. europaea* was grown in the calcium carbonate medium described. Subcultures (10% v/v) were made from actively growing cultures, either supplemented with no inhibitor, or with inhibitors at the concentrations used for the activity experiments (i.e., 10 µM ATU, 8 µM octyne, 200 and 400 µM PTIO). Cultures were set up in 120-ml serum bottles with silicone stoppers, and 25-ml volumes. Cultures were incubated in the dark, at 30ᵒC, and without shaking. All conditions were performed in triplicate.

**Supplementary Figures**


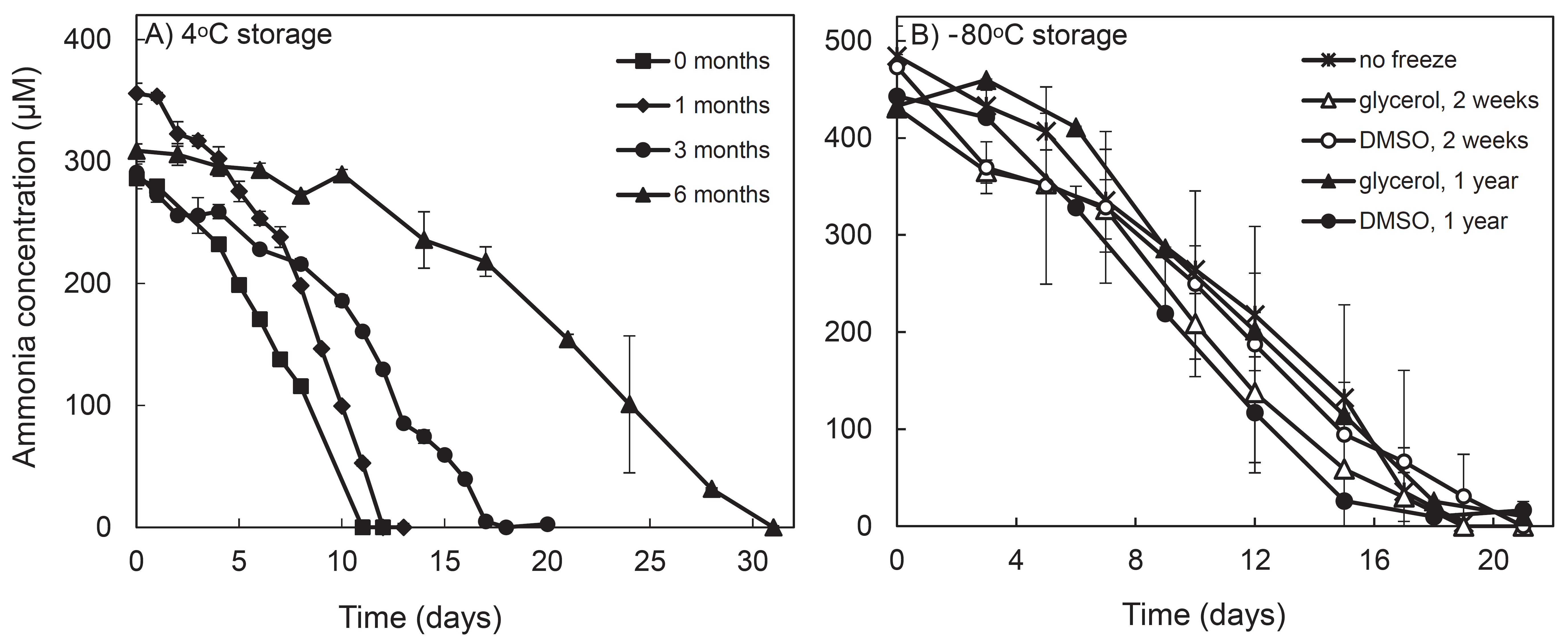


**Supplementary Figure S1.** Ammonia oxidation of *Candidatus* Nitrosocosmicus exaquare after storage in calcium carbonate-based growth media at A) 4°C, and B) -80°C in 35% glycerol or 7% DMSO. Times indicated in legends represent storage time in a given condition. Upon inoculation of stored cells into fresh media (10% inoculum for A; 5% inoculum for B), all cultures were incubated in the dark at 28°C without shaking. Error bars indicate standard error of the mean for biological duplicates.


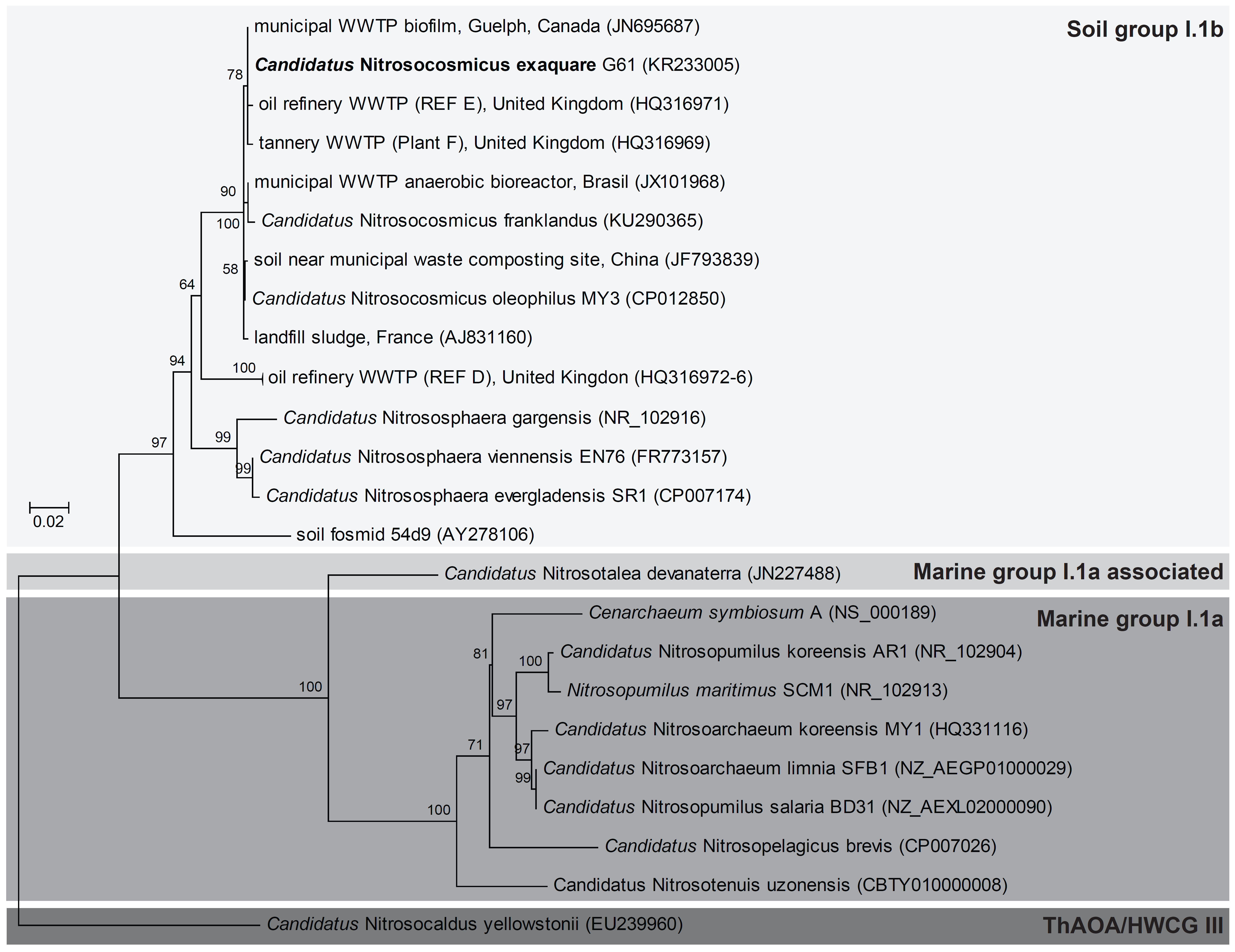


**Supplementary Figure S2.** Phylogenetic affiliations of the *Candidatus* Nitrosocosmicus exaquare 16S rRNA gene and those of other cultured *Thaumarchaeota* members and environmental sequences. This tree was inferred using the Maximum Likelihood method based on the General Time Reversible model. The tree is drawn to scale, with branch lengths measured as the number of substitutions per site. Bootstrap values are located above branches and are based on 500 replicates; only bootstrap values greater than 50% are indicated on tree. The scale bar represents 5% nucleotide divergence.


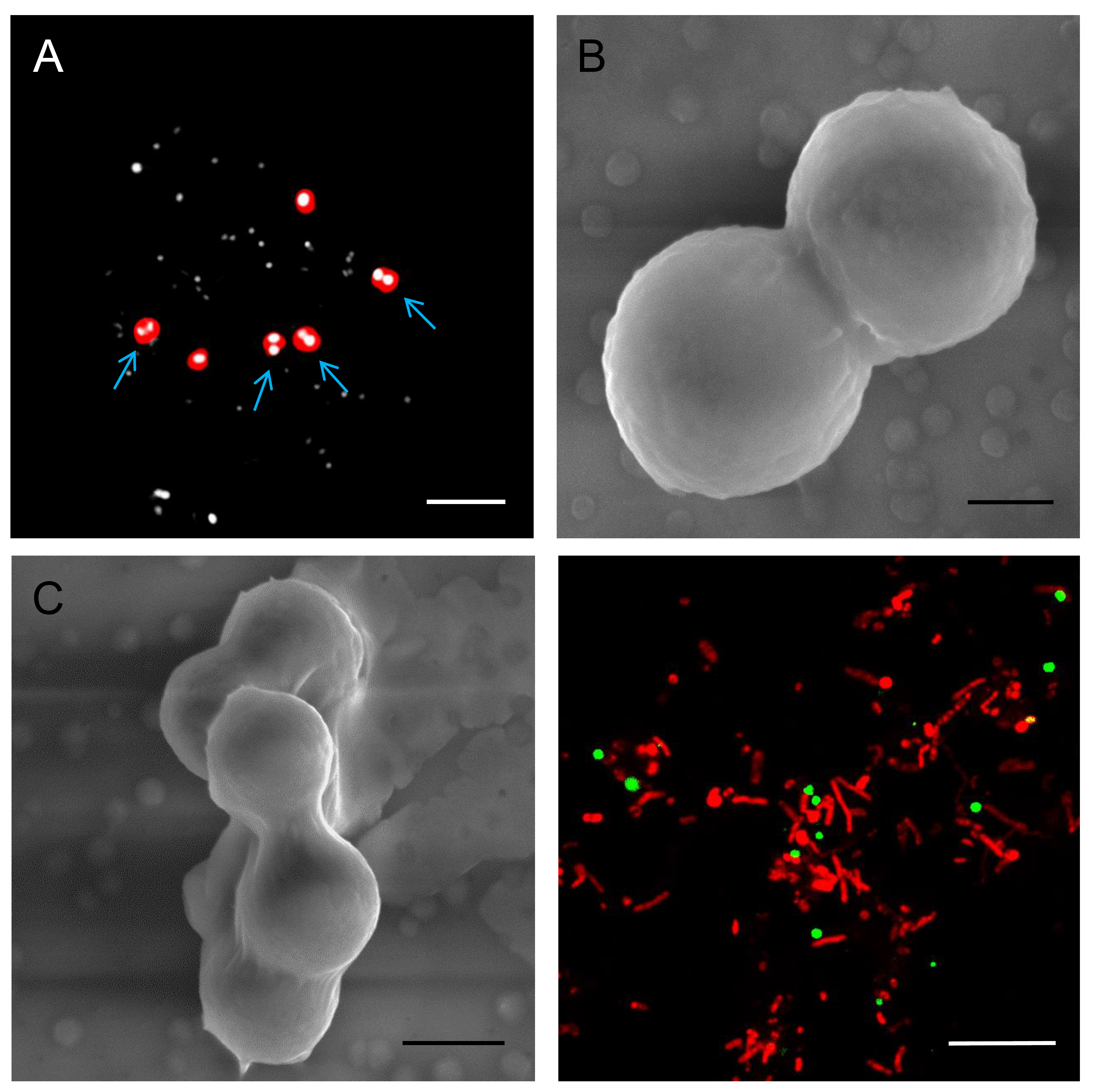


**Supplementary Figure S3.** Micrographs of *Candidatus* Nitrosocosmicus exaquare. A) CARD-FISH of *Ca.* N. exaquare cells in enrichment culture, labelled with probe Arch915 using Cy3 tyramides (red). DNA is stained non-specifically with DAPI (white). The arrows indicate cells containing two discrete regions of DNA. The scale bar represents 5 µM. B) Scanning electron micrograph of *Ca.* N. exaquare in pairs (scale bar 400 µm) and C) clusters (scale bar 1 µm). D) DOPE-FISH image of *Ca.* N. exaquare (green; Thaum726 double-labelled with fluorescein, applied with unlabelled competitor probes comp_thaum726A and comp_thaum726B) and bacteria (red; EUB338 mix double-labelled with Cy3) in RBC biofilm (scale bar 10 µm).

**Supplementary Figure S4.** Differential coverage binning of metagenomic contigs originating from *Ca*. N. exaquare enrichment cultures. Enrichment culture without supplemented organic carbon (G6) is shown on the x-axis, and media supplemented with 0.5 mM taurine is shown on the y-axis (G6T). All genomic DNA was extracted using the PowerSoil DNA Isolation Kit (MO BIO). This image and additional details are available at <http://madsalbertsen.github.io/mmgenome/>. Note that proportion of *Thaumarchaeota* differs from that reported based on qPCR because DNA for genome sequencing was extracted from enrichment cultures at an earlier time point.


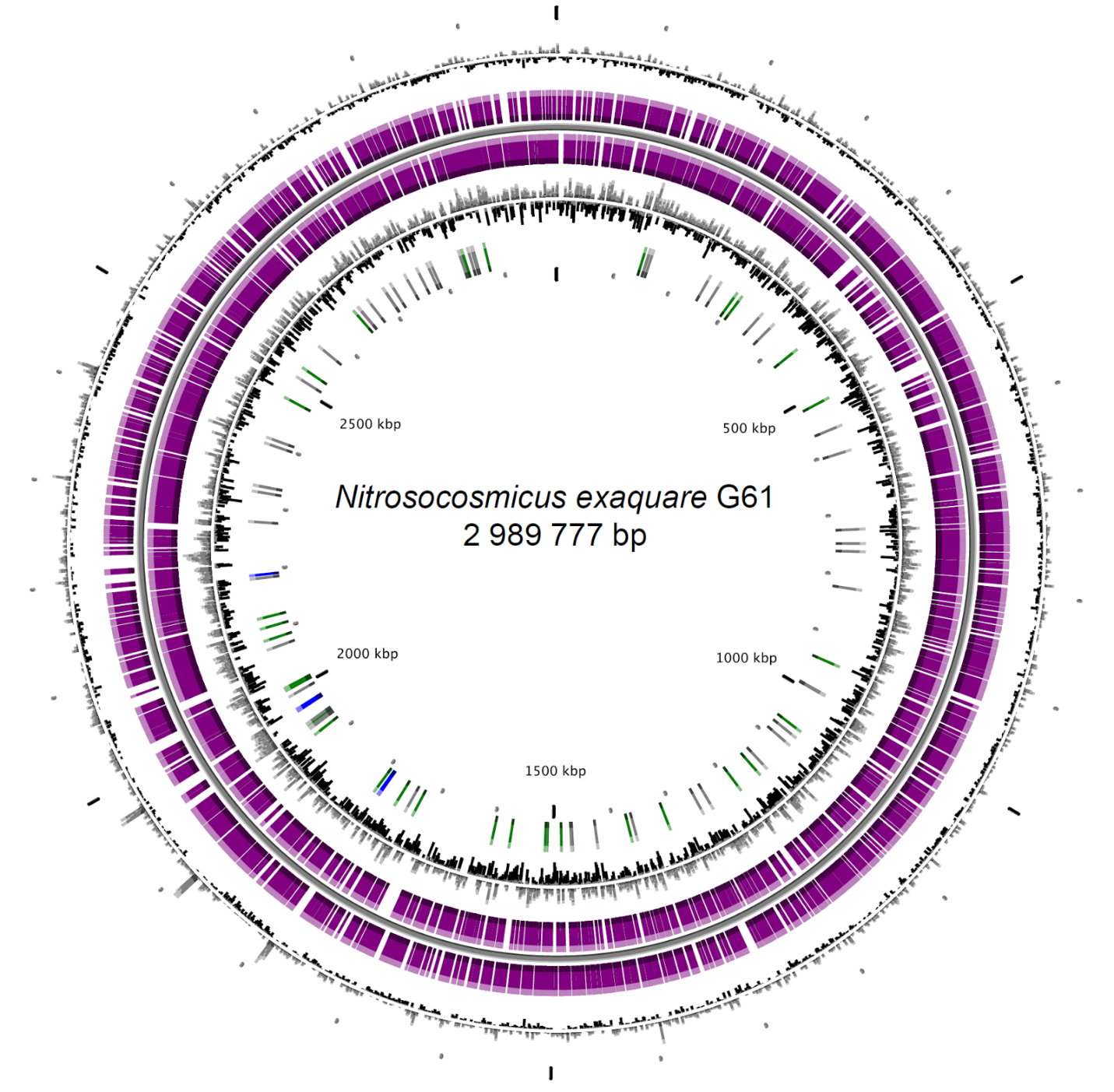


**Supplementary Figure S5.** Circular genome plot of *Candidatus* Nitrosocosmicus exaquare. Circles display (from the outside): (1) GC percent deviation (GC window - mean GC) in a 1000-bp window, (2) predicted coding sequences transcribed in the clockwise direction, and (3) predicted coding sequences transcribed in the counterclockwise direction. (4) GC skew (G+C/G-C) in a 1000-bp window, (5) rRNA (blue), tRNA (green), transposable elements (pink), and pseudogenes (grey).

**
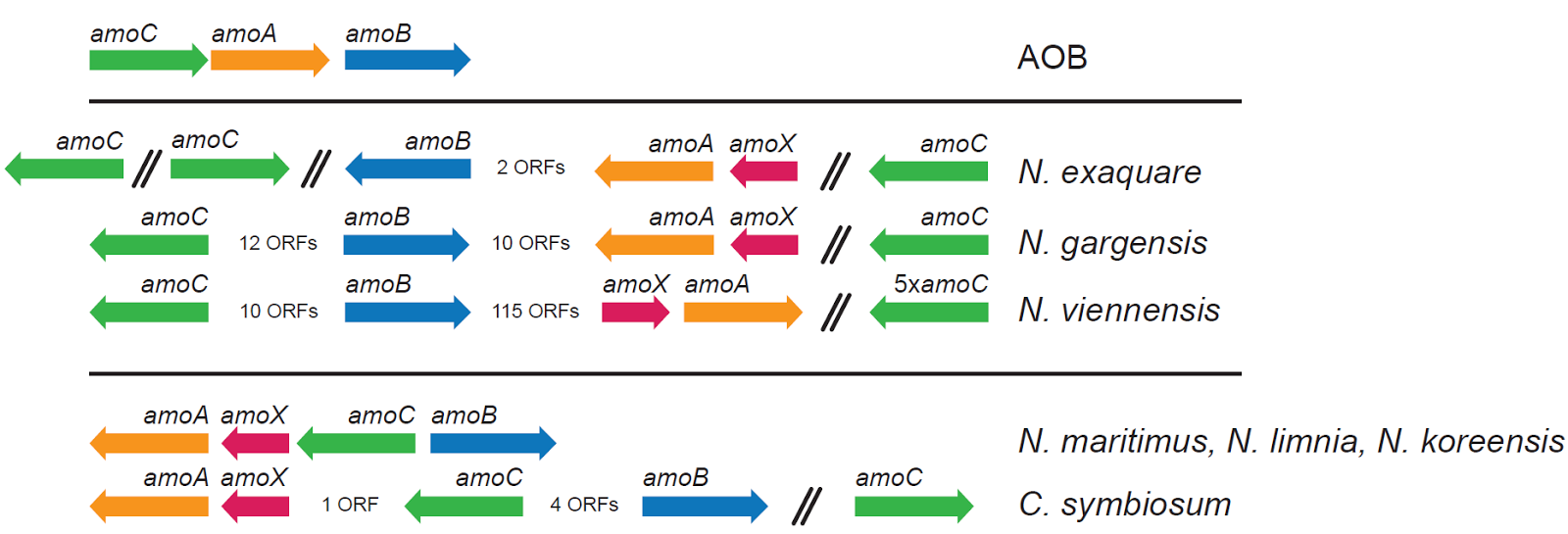
**

**Supplementary Figure S6.** Ammonia monooxygenase gene arrangement of *Candidatus* Nitrosocosmicus exaquare, selected group I.1b and I.1a *Thaumarchaeota*, and known ammonia-oxidizing bacteria (AOB). Slashes indicate large spatial separation of genes (>150 ORFs). *Candidatus* has been excluded from organism names for brevity.


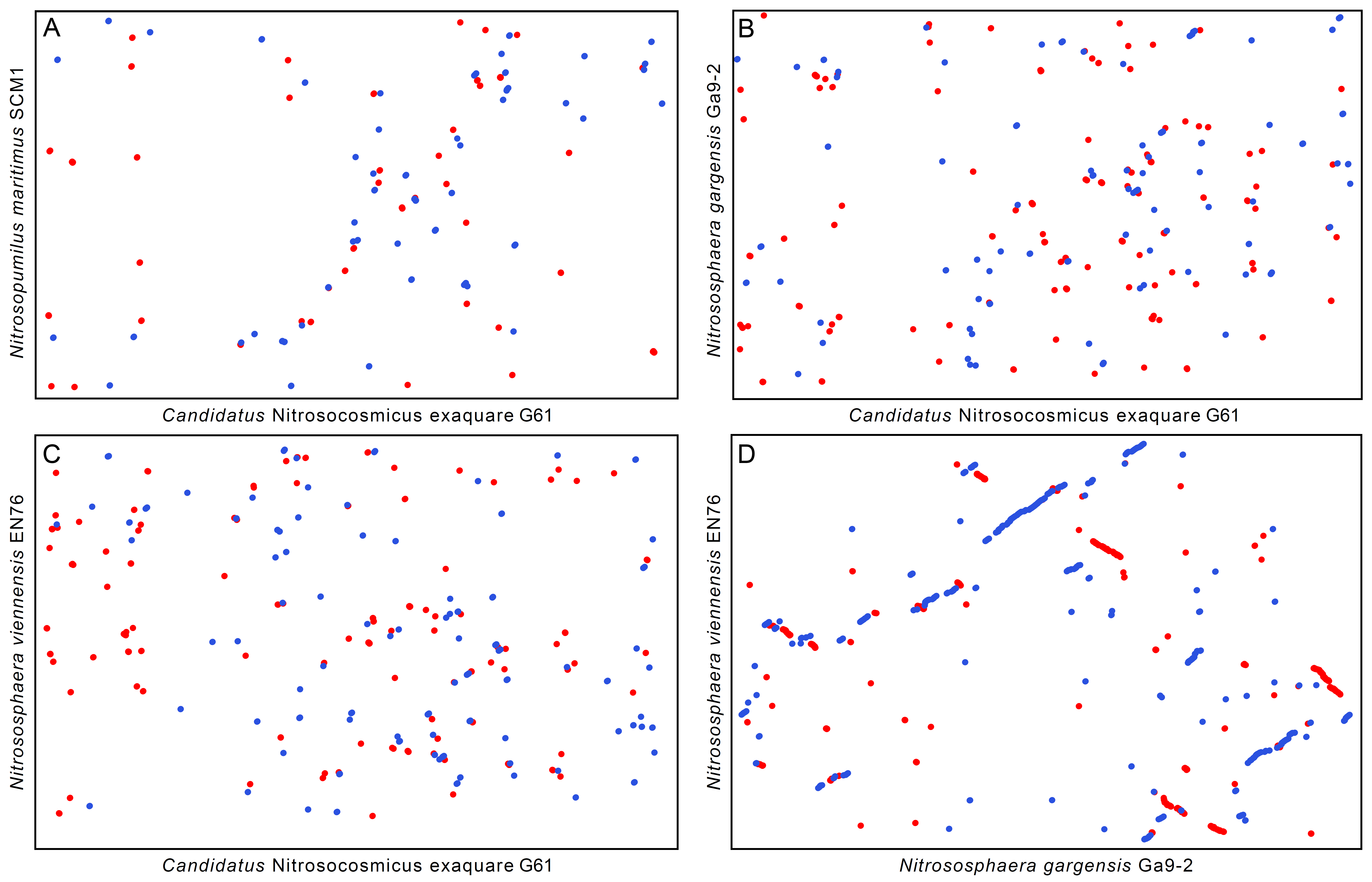


**Supplementary Figure S7.** Dot plot representation of pairwise alignments of *Ca.* N. exaquare*,* N. gargensis and *N. viennensis* genomes. Dot plots were generated in IMG/ER using PROmer, with alignments performed using six frame amino acid translations of the DNA input sequence. For all plots, a dot represents a match between the two genome sequences, with red and blue indicating forward and reverse matches, respectively.


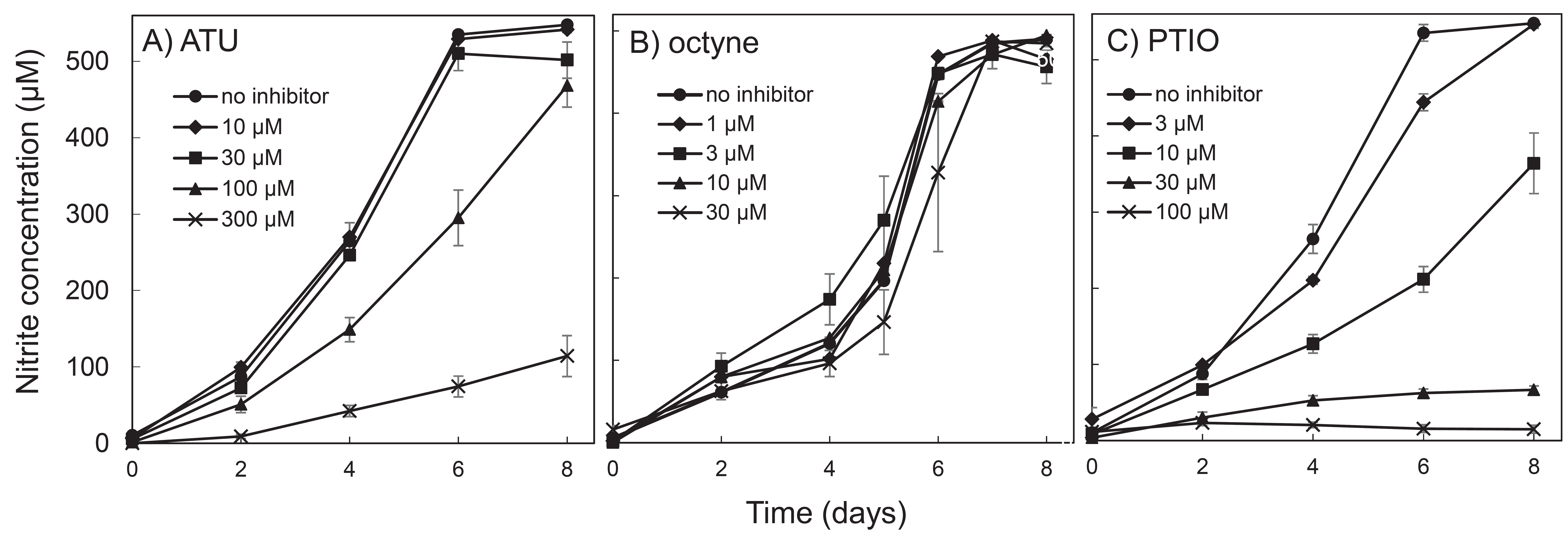


**Supplementary Figure S8.** Ammonia-oxidizing activity of *Candidatus* Nitrosocosmicus exaquare in the presence of nitrification inhibitors ATU, PTIO and octyne, following a 10% inoculum into fresh media containing 0.5 mM NH_4_Cl. Error bars represent the standard deviation of biological triplicates. Error bars not seen are contained within symbols.


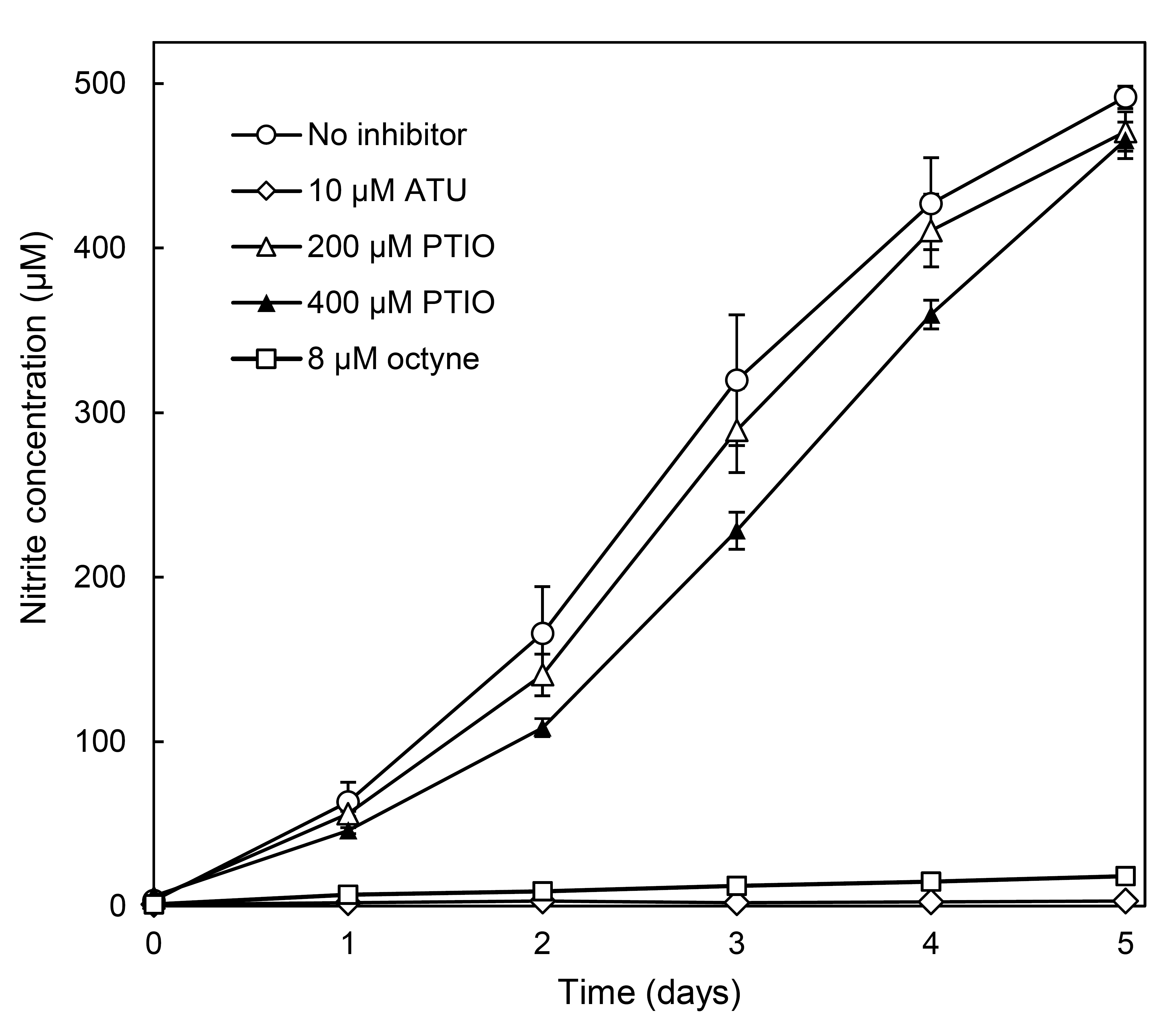


**Supplementary Figure S9.** *Nitrosomonas europaea* activity with inhibitors used in this study. All incubations were performed at the same time using the same inoculum. Error bars represent the standard error of triplicate incubations. Error bars not seen are contained within symbols.


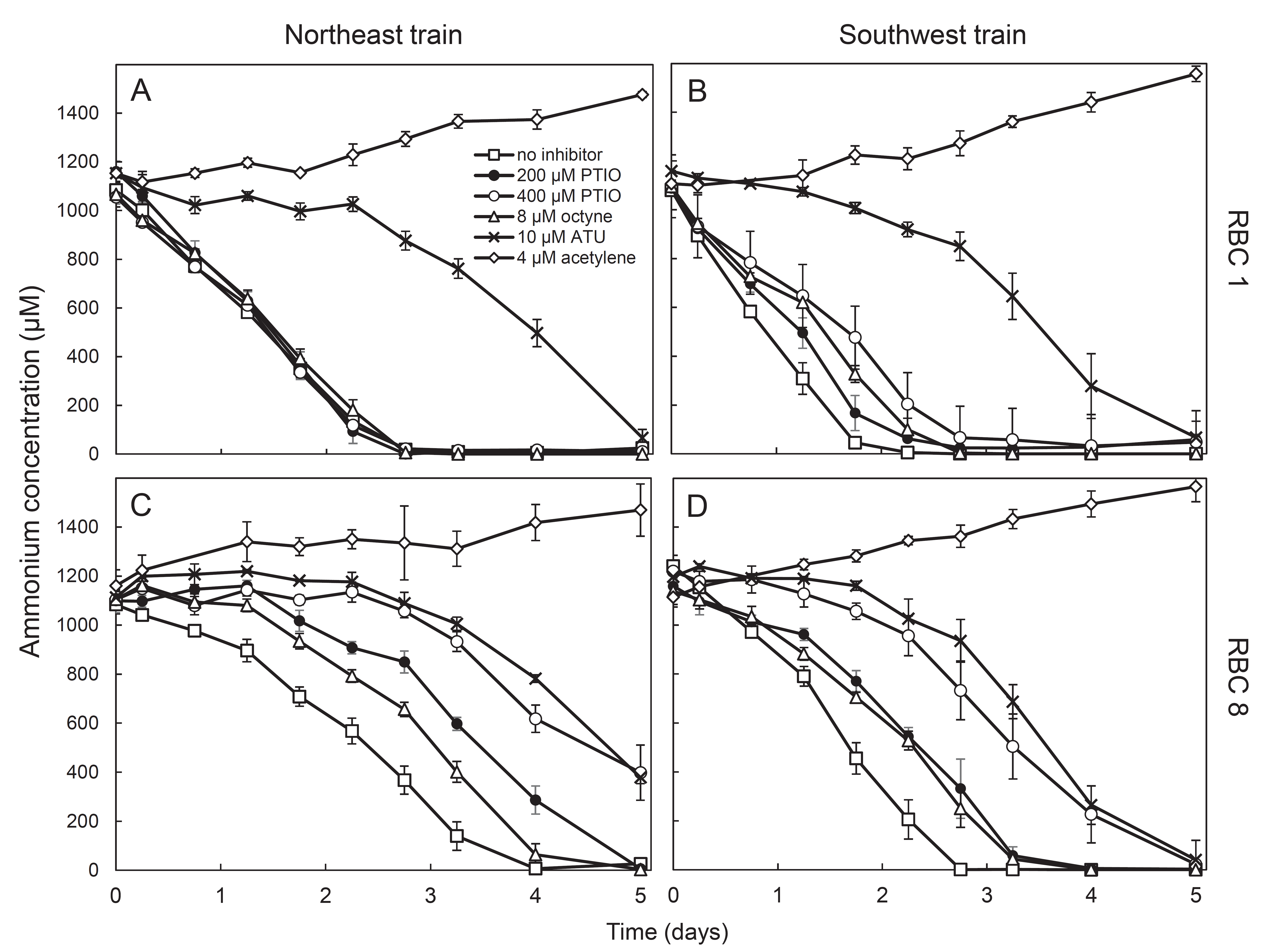


**Supplementary Figure S10.** Ammonia oxidation by biofilm incubated in wastewater influent supplemented with 1 mM NH_4_Cl. All incubations were performed at 25°C, in the dark, without shaking. Error bars indicate standard error of the mean for biological triplicates. Error bars not seen are contained within symbols.


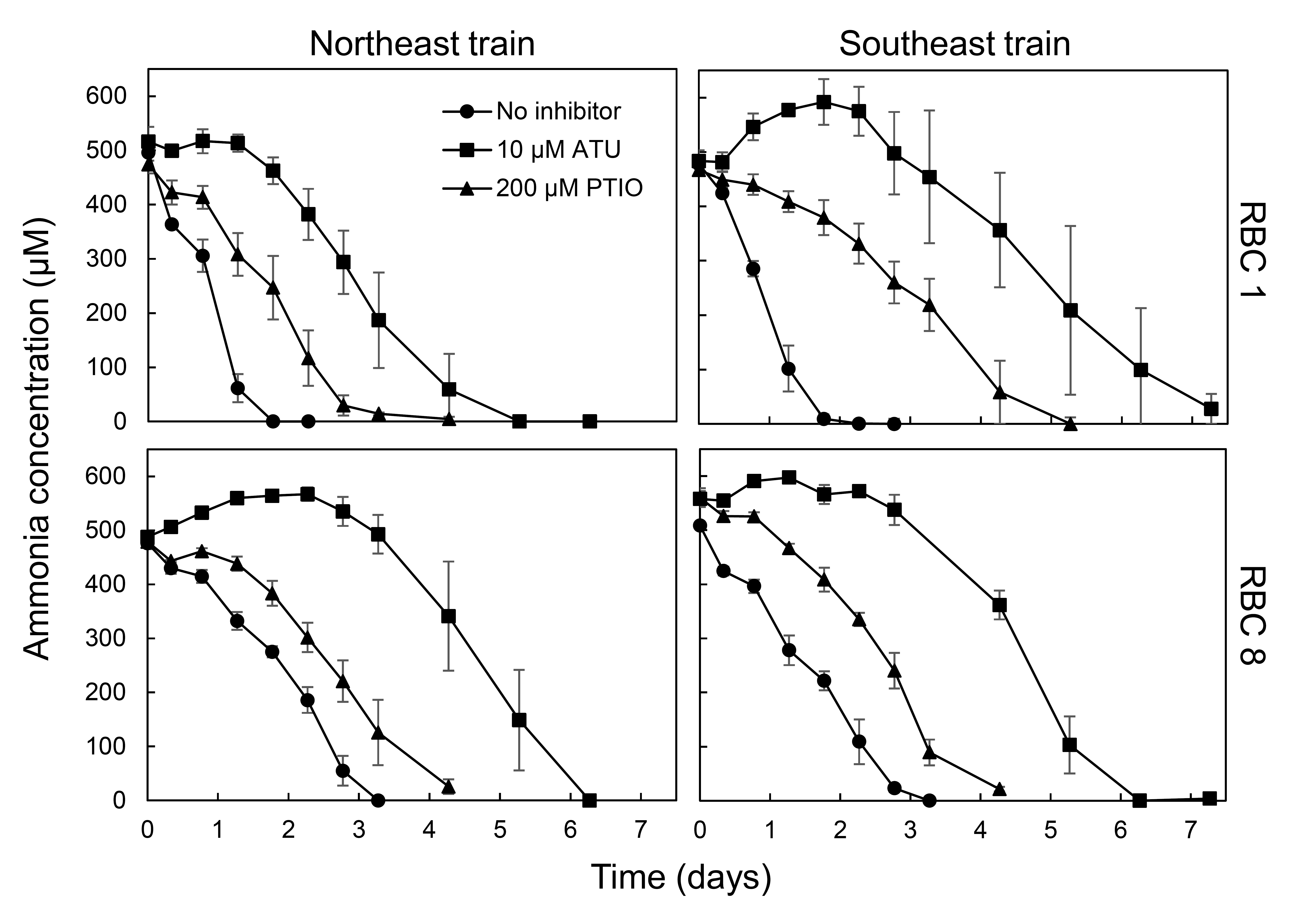


**Supplementary Figure S11.** Ammonia-oxidizing activity in Guelph RBC biofilm samples (April 2015; pre-cleaning). Incubations consisted of 1% biofilm (w/v) suspended in 0.22 μm-filtered RBC influent, supplemented with 0.5 mM NH_4_Cl. Error bars represent the standard error of triplicate incubations. Error bars not seen are contained within the symbols.

**Supplementary Tables**

**Supplementary Table S1. Guelph WWTP RBC biofilm sampling details**

| Collection date | RBCs sampled | Experiments/Analyses | Corresponding figures and tables |
| --- | --- | --- | --- |
| September 2012 | Southeast RBC 8 | Inoculum for cultivation | Described in text |
| April 2013 | Southeast RBC 1 and 8 | FISH  quantitative FISH | Fig. 3C, Fig. S3D  Described in text |
| July 2013 | Southeast RBC 1 and 8 | MAR-FISH | Fig. 7 |
| April 2015 | Northeast RBC 1 and 8  Southeast RBC 1 and 8 | Incubations with inhibitors  Biofilm regeneration | Fig. S11  Fig. S12 |
| December 2015 | Northeast RBC 1 and 8  Southwest RBC 1 and 8 | Incubations with inhibitors  qPCR | Fig. 6  Table S5 |

**Supplementary Table S2. Sequences of FISH probes used in this study**

| **Probe** | **Target** | **Sequence (5’ to 3’)** | **Reference** |
| --- | --- | --- | --- |
| Thaum726 | *Thaumarchaeota* (groups I.1a, I.1b, *Nitrosocaldales*, Group I.1d) | GCT TTC ATC CCT CAC CGT C | Beam, 2015 |
| Comp_thaum726A | competitor for thaum726 | GCT TTC GTC CCT CAC CGT C | Beam, 2015 |
| Comp_thaum726B | competitor for thaum726 | GCT TTC ATC CCT CAC TGT C | Beam, 2015 |
| Arch915 | most archaea | GTG CTC CCC CGC CAA TTC CT | Stahl and Amann, 1991 |
| EUB338 mix* | most bacteria | GCT GCC TCC CGT AGG AGT  GCA GCC ACC CGT AGG TGT  GCT GCC ACC CGT AGG TGT | Amann *et al.*, 1990; Daims *et al.*, 1999 |
| Ntspa662 | Genus *Nitrospira* | GGA ATT CCG CGC TCC TCT | Daims *et al.*, 2001 |
| Comp_Ntspa662 | Competitor for Ntspa662 | GGA ATT CCG CTC TCC TCT | Daims *et al.*, 2001 |
| Nit3 | Genus *Nitrobacter* | CCT GTG CTC CAT GCT CCG | Wagner *et al.*, 1995 |
| cNit3 | Competitor for NitC | CCT GTG CTC CAG GCT CCG | Wagner *et al.*, 1995 |
| Ntoga122 | Genus *Nitrotoga* | TCC GGG TAC GTT CCG ATA T | Lücker *et al.*, 2015 |
| c1Ntoga122 | Competitor for Ntoga122 | TCW GGG TAC GTT CCG ATA T | Lücker *et al.*, 2015 |
| c2Ntoga122 | Competitor for Ntoga122 | TCY GGG TAC GTT CCG ATG T | Lücker *et al.*, 2015 |
| NEU** | *N. europaea* & *N. eutropha* | CCC CTC TGC TGC ACT CTA | Wagner *et al.*, 1995 |
| CTE** | Competitor for NEU | TTC CAT CCC CCT CTG CCG | Wagner *et al.*, 1995 |
| Nso192** | *Nitrosomonas oligotropha* | CTT TCG ATC CCC TAC TTT CC | Adamczyk *et al.*, 2003 |
| Comp_Nso192** | Competitor for Nso192 | CTT TCG ATC CCC TGC TTC C | Adamczyk *et al.*, 2003 |
| Nso 1225** | Most beta-proteobacterial AOB | CGC CAT TGT ATT ACG TGT GA | Mobarry *et al.*, 1996 |
| nonsense | nonsense (negative control) probe | AGA GAG AGA GAG AGA GAG | Hatzenpichler *et al.*, 2008 |

*equimolar concentrations of EUB338, EUB338-II, and EUB338-III
**equimolar concentrations of these probes were used together to target AOB

**Supplementary Table S3. Heterotrophic bacteria sequenced from *Ca*. N. exaquare enrichment culture**

| Bin | Class | Order | Family | Genus | IMG ER genome ID^ǂ^ |
| --- | --- | --- | --- | --- | --- |
| 2 | *Alphaproteobacteria* | *Sphingomonadales* | *Sphingomonadaceae* | *Sphingomonas* | 2606217231 |
| 3 | *Alphaproteobacteria* | *Sphingomonadales* | *Sphingomonadaceae* | *Sphingopyxis* | 2606217232 |
| 4 | *Alphaproteobacteria* | *Rhizobiales* | *Phyllobacteriaceae* | *Mesorhizobium* | 2606217233 |
| 5 | *Alphaproteobacteria* | *Rhodospirillales* | *Reyranella* | n.c. | 2606217234 |
| 6 | *Alphaproteobacteria* | *Rhizobiales* | *Bradyrhizobiaceae* | *Bradyrhizobium* | 2606217235 |
| 7 | *Sphingobacteria* | *Sphingobacteriales* | *Chitinophagaceae* | *Flavitalea* | 2606217236 |
| 8 | *Gemmatimonadetes* | *Gemmatimonadales* | *Gemmatimonadaceae* | *Gemmatimonas* | 2606217237 |

^ǂ^ genomes publicly available on IMG ER platform

n.c.: no classification at this level

**Supplementary Table S4. Average amino acid identities (AAIs) of *Ca*. N. exaquare and selected AOA representatives**

| Organism | AAI (%) |
| --- | --- |
| *Nitrosopumilus maritimus* SCM1 | 46.0 |
| *Nitrosotalea devanaterra* Nd1 | 47.6 |
| *Nitrosotenuis uzonensis* N4 | 46.9 |
| *Nitrosotenuis cloacae* SAT1 | 46.3 |
| *Nitrososphaera gargensis* Ga9-2 | 52.6 |
| *Nitrososphaera viennensis* EN76 | 52.0 |
| *Nitrososphaera evergladensis* SR1 | 52.0 |

**Supplementary Table S5. Quantitative PCR data from Guelph WWTP biofilm samples (December 2015)**

| Biofilm Sample | AOA 16S rRNA genes* | SD | AOB *amoA* genes* | SD | Bacterial 16S rRNA genes* | SD | Total AOP (AOA 16S +  AOB *amoA* genes) | % AOA of total AOP | % AOA of TC (total 16S rRNA genes) |
| --- | --- | --- | --- | --- | --- | --- | --- | --- | --- |
| NE RBC 1 | 5689 | 249 | 4750 | 492 | 144883 | 12041 | 10440 | 54.5 | 3.78 |
| NE RBC 8 | 2682 | 96 | 335 | 96 | 194541 | 1858 | 3017 | 88.9 | 1.36 |
| SW RBC 1 | 2789 | 322 | 1970 | 46 | 190359 | 1187 | 4759 | 58.6 | 1.44 |
| SW RBC 8 | 1343 | 165 | 421 | 74 | 141691 | 1605 | 1764 | 76.2 | 0.94 |

*copies ng^-1^ genomic DNA (gene copies not standardized based on 16S rRNA gene copy number)

SD: standard deviation of technical duplicates

AOP: ammonia-oxidizing prokaryotes

TC: total community (total bacterial and thaumarchaeotal 16S rRNA genes)

**References**

Adamczyk J, Hesselsoe M, Iversen N, Horn M, Lehner A, Nielsen PH, *et al.* (2003). The isotope array, a new tool that employs substrate-mediated labeling of rRNA for determination of microbial community structure and function. *Appl Environ Microbiol* **69**:6875–6887.

Albertsen M, Hugenholtz P, Skarshewski A, Nielsen KL, Tyson GW, Nielsen PH. (2013). Genome sequences of rare, uncultured bacteria obtained by differential coverage binning of multiple metagenomes. *Nat Biotechnol* **31**:533–538.

Amann RI, Binder BJ, Olson RJ, Chisholm SW, Devereux R, Stahl DA. (1990). Combination of 16S rRNA-targeted oligonucleotide probes with flow cytometry for analyzing mixed microbial populations. *Appl Environ Microbiol* **56**:1919–1925.

Beam JP. (2015). Geobiological interactions of archaeal populations in acidic and alkaline geothermal springs of Yellowstone National Park, WY, USA. Montana State University.

Daims H, Brühl A, Amann R, Schleifer KH, Wagner M. (1999). The domain-specific probe EUB338 is insufficient for the detection of all Bacteria: development and evaluation of a more comprehensive probe set. *Syst Appl Microbiol* **22**:434–444.

Daims H, Nielsen JL, Nielsen PH, Schleifer KH, Wagner M. (2001). *In situ* characterization of *Nitrospira*-like nitrite-oxidizing bacteria active in wastewater treatment plants. *Appl Environ Microbiol* **67**:5273–5284.

Dupont CL, Rusch DB, Yooseph S, Lombardo M-J, Richter RA, Valas R, *et al.* (2012). Genomic insights to SAR86, an abundant and uncultivated marine bacterial lineage. *ISME J* **6**:1186–1199.

Goris J, Konstantinidis KT, Klappenbach JA, Coenye T, Vandamme P, Tiedje JM. (2007). DNA-DNA hybridization values and their relationship to whole-genome sequence similarities. *Int J Syst Evol Microbiol* **57**:81–91.

Hatzenpichler R, Lebedeva EV, Spieck E, Stoecker K, Richter A, Daims H, *et al.* (2008). A moderately thermophilic ammonia-oxidizing crenarchaeote from a hot spring. *Proc Natl Acad Sci USA* **105**:2134–2139.

Herbold CW, Lebedeva EV, Palatinszky M. (2016). *Candidatus* Nitrosotenuis. In:*Bergey’s Manual of Systematics of Archaea and Bacteria*, William B. Whitman (ed), John Wiley & Sons Ltd.: Chichester, England, *In press*.

Huson DH, Mitra S, Ruscheweyh H-J, Weber N, Schuster SC. (2011). Integrative analysis of environmental sequences using MEGAN4. *Genome Res* **21**:1552–1560.

Hyatt D, Chen G-L, Locascio PF, Land ML, Larimer FW, Hauser LJ. (2010). Prodigal: prokaryotic gene recognition and translation initiation site identification. *BMC Bioinformatics* **11**:119.

Konstantinidis KT, Tiedje JM. (2005). Towards a genome-based taxonomy for prokaryotes. *J Bacteriol* **187**:6258–6264.

Kurtz S, Phillippy A, Delcher AL, Smoot M, Shumway M, Antonescu C, *et al.* (2004). Versatile and open software for comparing large genomes. *Genome Biol* **5**:R12.

Lücker S, Schwarz J, Gruber-Dorninger C, Spieck E, Wagner M, Daims H. (2015). *Nitrotoga*-like bacteria are previously unrecognized key nitrite oxidizers in full-scale wastewater treatment plants. *ISME J* **9**:708–720.

Mobarry B, Wagner M, Urbain V, Rittmann B, Stahl D. (1996). Phylogenetic probes for analyzing abundance and spatial organization of nitrifying bacteria. *Appl Environ Microbiol* **62**:2156–2162.

Rodriguez LM, Konstantinidis KT. (2014). Bypassing cultivation to identify bacterial species. *Microbe* **9**:111–118.

Rodriguez-R LM, Konstantinidis KT. (2016). The enveomics collection: a toolbox for specialized analyses of microbial genomes and metagenomes. *Peer J Prepr* **4**:e1900v1.

Stahl DA, Amann R. (1991). Development and application of nucleic acid probes in bacterial systematics. In:*Nucleic Acid Techniques in Bacterial Systematics‏*, Stackebrandt, E & Goodfellow, M (eds), John Wiley & Sons Ltd.: Chichester, England, pp. 205–248.

Tourna M, Stieglmeier M, Spang A, Könneke M, Schintlmeister A, Urich T, *et al.* (2011). *Nitrososphaera viennensis*, an ammonia oxidizing archaeon from soil. *Proc Natl Acad Sci USA* **108**:8420–8425.

Wagner M, Rath G, Amann R, Koops H-P, Schleifer K-H. (1995). *In situ* identification of ammonia-oxidizing bacteria. *Syst Appl Microbiol* **18**:251–264.
